# Chronic Stress Exacerbates Cerebral Amyloid Angiopathy Through Promoting Neutrophil Extracellular Traps Formation

**DOI:** 10.1002/advs.202404096

**Published:** 2024-09-26

**Authors:** Huipeng Huang, Xiaohui Deng, Yuge Wang, Shishi Shen, Shisi Wang, Mengyan Hu, Sanxin Liu, Xiaotao Su, Chunyi Li, Tiemei Li, Zhengqi Lu, Wei Cai

**Affiliations:** ^1^ Department of Neurology Mental and Neurological Disease Research Center The Third Affiliated Hospital of Sun Yat‐sen University Guangzhou 510630 China; ^2^ Guangdong Provincial Key Laboratory of Brain Function and Disease Guangzhou 510630 China

**Keywords:** cerebral amyloid angiopathy, chronic stress, neutrophil extracellular traps, norepinephrine

## Abstract

Cerebral amyloid angiopathy (CAA) is the leading cause of vascular dementia among the elderly. Neuropsychiatric symptoms are commonly manifested in cerebral amyloid angiopathy patients but are usually considered as consequences of cerebral amyloid angiopathy pathology. Here, it is reported that chronic stress promotes cerebral amyloid angiopathy progression, which enhances deposition of amyloid protein beta (Aβ) in brain blood vessels and exacerbates subsequent brain injury. Mechanistically, neutrophil is implicated in cerebral amyloid angiopathy development. Aβ that accumulates in brain vasculature induces neutrophil extracellular traps (NETs) by activating STAT6 signaling, which inhibits neutrophil apoptosis and switches the programmed cell death toward NETosis. During chronic stress, circulatory Norepinephrine (NE) strengthens STAT6 activation in neutrophil and promotes NET formation, thus exacerbates the NET‐dependent angiopathy. It is demonstrated that inhibiting neutrophil chemotaxis towards brain or suppressing NET formation both ameliorate cerebral amyloid angiopathy severity in the context of chronic stress. Therefore, it is proposed that stress‐associated psychological disorders and NETs are promising therapeutic targets in cerebral amyloid angiopathy.

## Graphical abstract

Neutrophils target towards brain in CAA and release NETs that cause blood vessel damage and subsequent brain injury, which is exacerbated by chronic stress, through increasing circulatory Norepinephrine (NE) to promote NET formation, thus accelerating the NET‐dependent angiopathy in CAA. Inhibiting neutrophil chemotaxis or suppressing NET formation both ameliorate CAA severity in the context of chronic stress.

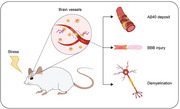

## 1 Introduction

Cerebral amyloid angiopathy (CAA), which is characterized with deposition of amyloid protein beta (Aβ) in brain vasculature, is the leading cause of vascular dementia and an important comorbidity of Alzheimer's disease (AD).^[^ ^1^ ^]^ Despite the high prevalence of CAA among the elderly,^[^ ^2^ ^]^ underlying mechanisms of CAA development remain largely elusive, which results in the lack of effective treatment.

Neuropsychiatric symptoms are commonly manifested in CAA. It is reported that depression and anxiety are evident in ≈48.8% and ≈32.6% of CAA patients respectively.^[^ ^3^ ^]^ Generally, psychological abnormalities are considered as the result of CAA pathology.^[^ ^4^, ^5^, ^6^ ^]^ However, whether psychological disorders contribute to CAA development in reverse is less concern. As a result, the therapeutic significance of tackling neuropsychiatric symptoms in CAA is usually underestimated. Chronic stress is a major risk factor of psychological disorders.^[^ ^7^, ^8^ ^]^ Accumulative evidence suggest that chronic stress promotes the progression of cancer^[^ ^9^ ^]^ and cardiovascular disorders.^[^ ^10^ ^]^ The impacts of chronic stress on CAA outcomes remain to be studied.

Neutrophil is the most abundant immune cell in the circulation which expresses stress‐associated neurotransmitter receptors including α‐ and β‐adrenergic receptors.^[^ ^11^, ^12^ ^]^ According to previous reports, neutrophil is activated in both stress‐stimulated animal models^[^ ^13^ ^]^ and patients with stress‐associated psychological disorders.^[^ ^14^, ^15^ ^]^ Among the pro‐inflammatory mediators produced by activated neutrophils, neutrophil extracellular traps (NETs) exert the most destructive effects.^[^ ^16^ ^]^ On one hand, reactive oxygen species (ROS) and proteases in NETs directly cause tissue damage.^[^ ^17^ ^]^ On the other hand, NET represents a danger‐associated molecular pattern (DAMP),^[^ ^18^ ^]^ which attracts the infiltration of other immune cells and subsequently exacerbates the inflammatory injury in lesion.^[^ ^19^ ^]^ Therefore, we infer that neutrophil which is activated during chronic stress and its product of NET could promote CAA progression.

One key regulatory mechanism of NETosis involves the transcription factor Signal Transducer and Activator of Transcription 6 (STAT6), a transcription factor that plays a crucial role in immune response regulation. Dysregulation of STAT6 signaling contributes to excessive NETs formation,^[^ ^20^, ^21^ ^]^ which can exacerbate inflammation and consequently tissue damage in diseases where NETosis is a pathological feature.

In the current study, we report that chronic stress exacerbates amyloid angiopathy, blood‐brain barrier (BBB) injury, and myelin loss in CAA. Chronic stress provokes neutrophil chemotaxis towards the brain and promotes NET formation in Aβ‐deposited brain vasculature. Mechanistically, the amyloid peptide 1–40 (Aβ40) activates STAT6 signaling in neutrophils, which switches the programmed cell death from apoptosis towards NETosis. An increment of circulatory Norepinephrine (NE) during stress further enhances STAT6 activation and aggravates the NET‐dependent damage. We demonstrate that inhibiting neutrophil chemotaxis towards brain or suppressing NET formation both ameliorate CAA severity.

## 2 Results

### 2.1 Chronic Stress Provokes Neutrophil Chemotaxis Towards Brain Vasculature and Exacerbates CAA Outcomes

**Figure 1:**
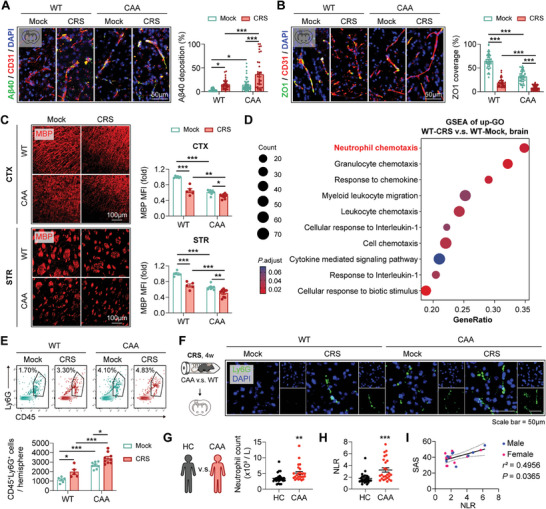
Chronic stress exacerbates CAA outcomes and brain‐targeting chemotaxis of neutrophil. A–F) WT or CAA mice (age = 12w) were stimulated with chronic restraint stress (CRS) for 4 weeks. A–C) Coronal brain sections were subjected to immunostaining of indicated markers. *N* = 5–9 mice in each group. A) Coverage of Aβ40 fluorescence in the range of blood vessels (outlined with CD31) in cortex was calculated. A total of 33–43 blood vessels in each group were analyzed respectively. ^*^ *p* < 0.05, ^***^ *p* < 0.001; by one‐way *ANOVA*. B) Coverage of ZO1 fluorescence in the range of blood vessels (outlined with CD31) in cortex was calculated. A total of 39–41 blood vessels in each group were analyzed respectively. ^*^ *p* < 0.05, ^***^ *p* < 0.001; by one‐way *ANOVA*. C) MBP MFI in cortex (CTX) and striatum (STR) was calculated. *N* = 5–9 mice in each group. ^*^ *p* < 0.05, ^**^ *p* < 0.01, ^***^ *p* < 0.001; by one‐way *ANOVA*. D) Brain tissue of WT‐Mock and WT‐CRS mice was subjected to bulk RNAseq. Up‐regulated differential expressed genes (DEGs) in WT‐CRS brain compared with WT non‐stimulated brain were analyzed with gene ontology (GO) (up‐GO). The result of further gene set enrichment analysis (GSEA) with the up‐GO was displayed. *N* = 2 mice in each group. E) Neutrophil count per hemisphere was quantified with flow cytometry. Representative flow plots with the percentage of neutrophils among brain singlets were displayed. *N* = 5–9 mice in each group. ^*^ *p* < 0.05, ^***^ *p* < 0.001; by one‐way *ANOVA*. F) Neutrophil infiltration in brain was confirmed with immunostaining of Ly6G (green) and subsequent confocal microscopy. Experiments were repeated for 3 times and representative images (cortex) were displayed. G,H) Circulatory neutrophil count G) and neutrophil‐lymphocyte ratio (NLR) H) in CAA patients and healthy controls (HC) were quantified. *N* = 27 in HC group. *N* = 26 in CAA groups. ^***^ *p* < 0.001; by two‐tailed Student's *t‐*test. I) Association of SAS score and NLR in CAA patients was evaluated with Spearman correlation analysis. *N* = 18. Blue dots indicated men (*N* = 9) while pink dots indicated women (*N* = 9).

To study the impacts of chronic stress on CAA progression, the CAA murine models of homozygous Tg‐SwDI/B mice and the wild type (WT) controls (12w of age, a critical stage when Aβ deposition initially primes as previously reported^[^ ^22^ ^]^) were subjected to chronic restraint stress (CRS) for 4w. Through behavior tests, we ensured mice of both sexes received CRS exhibited anxiety‐like behaviors (Figure S1A–C, Supporting Information). With immunostaining, we found that CAA models with CRS stimulation (CAA‐CRS) displayed deteriorated Aβ40 deposition in brain blood vessels (**Figure** **1**A; Figure S2A, Supporting Information), decreased tight junction protein expression (Figure 1B), severe myelin loss (Figure 1C). Given CAA mice begin to exhibit cognitive deficits at 4 months of age,^[^ ^22^ ^]^ we evaluated their cognition by Novel object recognition and Mirror water maze. Our assessments identified a significant exacerbation of cognitive impairment of CAA mice after CRS (Figure S3, Supporting Information). To explore the underlying mechanisms of chronic stress‐induced injury, brain tissue of mice with or without CRS simulation was subjected to bulk RNA sequencing (RNAseq). Gene set enrichment analysis (GSEA) with the up‐regulated gene ontology (GO) in the CRS‐stimulated mice revealed that neutrophil chemotaxis towards brain was enhanced (Figure 1D). With flow cytometric analysis (Figure 1E) and immunostaining (Figure 1F), we confirmed that the number of neutrophils in brain tissue was increased in both CAA‐ and WT‐CRS models compared with their non‐stimulated littermates, and both male and female mice exhibited increased number of neutrophils in brain tissue after receiving CRS (Figure S1D, Supporting Information) At the meantime, peripheral neutrophils were mobilized in both WT‐ and CAA‐CRS mice (Figure S2B, Supporting Information). Furthermore, CAA patients (Table S1, Supporting Information) displayed increased neutrophil count (Figure 1G) and neutrophil‐lymphocyte ratio (NLR) (Figure 1H) in blood tests when compared to age‐ and sex‐matched healthy controls (HC). Notably, NLR was positively correlated with the score of the Self‐rating Anxiety Scale (SAS) in patients (Figure 1I). Therefore, we hypothesized that neutrophils mediated the disease‐exacerbating effects of chronic stress in CAA.

**Figure 2:**
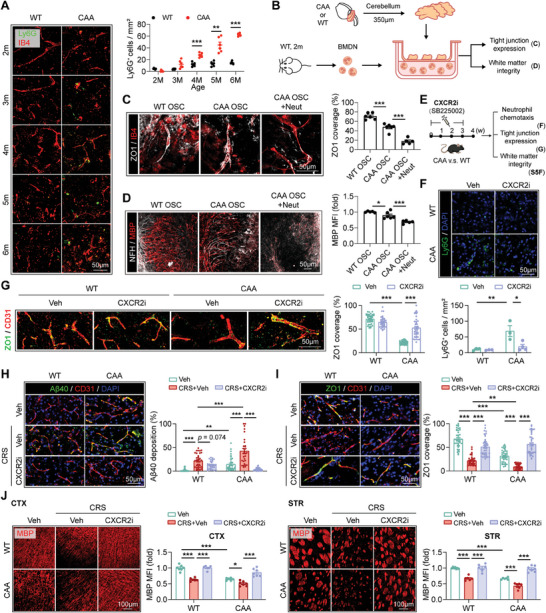
Neutrophils are implicated in CAA pathology. A) WT or CAA mice were sacrificed at indicated age and coronal brain sections were subjected to Ly6G and IB4 staining. Representative images showing neutrophil accumulation in cortex are displayed. *N* = 6 for each time point. ^**^ *p* < 0.01, ^***^ *p* < 0.001; by two‐way *ANOVA*. B–D) Organotypic slice cultures (OSCs) were prepared with the cerebellum of CAA or WT mice. BMDN were extracted from WT mice then cocultured with the OSCs through transwell for 4 h (5 × 10^4^ BMDN per culture system with three brain slices). Experiments were repeated for 5 times. B) Experimental design. C) ZO1 and IB4 were co‐stained to evaluate the tight junction loss of OSC after co‐cultured with BMDN. ^***^ *p* < 0.001; by one‐way *ANOVA*. D) White matter integrity was assessed with immunostaining of MBP and NFH. ^*^ *p* < 0.05, ^***^ *p* < 0.001; by one‐way *ANOVA*. E–G) CAA or WT mice (age = 12w) were treated with CXCR2i (SB225002, i.p., 2 mg k^−1^g, 3 weeks, 6 days each) and sacrificed at 4w after treatment onset. An equal volume of PBS (vehicle, Veh) was injected to the control groups. *N* = 3–4 in each group. ^*^ *p* < 0.05, ^**^ *p* < 0.01, ^***^ *p* < 0.001; by one‐way *ANOVA*. E) Experimental design. F) The efficacy of inhibition against brain‐targeting chemotaxis of neutrophil was verified by immunostaining of Ly6G (green). G) Expression of tight junction protein (ZO1^+^) on blood vessels (CD31^+^) was assessed with immunostaining. ZO1 coverage in a total of 34–40 blood vessels in each group was analyzed respectively. H,I) CAA and WT mice (age = 12w) were subjected to CRS for 4w with or without CXCR2i treatment (SB225002, i.p., 2 mg k^−1^g, 3 weeks, 6 days each). An equal volume of PBS (vehicle, Veh) was injected to the control groups. *N* = 6–7 in each group. ^*^ *p* < 0.05, ^**^ *p* < 0.01, ^***^ *p* < 0.001; by one‐way *ANOVA*. H) Aβ40 deposition along blood vessels (CD31^+^) as assessed with immunostaining. Aβ40 coverage in 30–38 blood vessels in each group was analyzed respectively. I) Tight junction (ZO1^+^) expression along blood vessels (CD31^+^) as assessed with immunostaining. ZO1 coverage in 36–42 blood vessels in each group was analyzed respectively. J) White matter integrity in cortex (CTX) and striatum (STR) was assessed with MBP staining.

To test the hypothesis, we first explored the temporal and spatial dynamics of neutrophils in CAA models. With immunostaining, we observed that CAA models displayed pronounced neutrophil accumulation in brain, which started from 3 to 4 months and increased along with age (**Figure** **2**A). Interestingly, neutrophils in CAA brains were largely confined within blood vessels at early age (3–4m) but intruded into brain parenchyma at later age (≥5m) (Figure 2A). Single‐cell RNA sequencing of CAA brains (age = 6m, a stage of more pronounced cognitive impairment that we believed to be associated with significant NVU alteration^[^ ^22^ ^]^) also revealed increasing number of neutrophil, and GO analysis of neutrophil revealed up‐regulation of binding, GO analysis of endothelial cell and GSEA analysis of pericyte revealed enhanced neutrophil chemotaxis (Figure S4, Supporting Information). Besides, the RNAseq data revealed increased expression of neutrophil‐attracting chemokines in CAA brains (Figure S5B,C, Supporting Information), which is ligand of CXCR2, the vital neutrophil chemotaxic driver. In accordance, neutrophil in the periphery was mobilized (Figure S5D,E, Supporting Information). The data illustrates that neutrophils migrate from periphery towards brain lesion in CAA.

We then evaluated the pathophysiological roles of neutrophils in CAA. In ex vivo experiments, brain organotypic slice cultures (OSCs) were prepared with CAA mice upon transwell then cocultured with primary bone marrow‐derived neutrophils (BMDN) from WT mice to mimick the perivascular pathway through which neutrophils can exert effects on the BBB and white matter without direct contact with brain tissue. (Figure 2B). We found that BMDN caused pronounced tight junction decrement (Figure 2C) and myelin loss (Figure 2D) in CAA OSCs. In vivo, neutrophil chemotaxis towards brain in CAA models was prohibited by inhibiting CXCR2, the key mobilization driver of neutrophils (Figure 2E,F; Figure S6, Supporting Information). Upon administration of the CXCR2 inhibitor

SB225002 (CXCR2i, i.p., 2 mg k^−1^g, 3w, 6 days each), tight junction protein expression (Figure 2G), white matter integrity (Figure S5F, Supporting Information) and cognition (Figure S8, Supporting Information) in CAA models were improved. These results indicate that neutrophil plays detrimental roles in CAA progression.

We thus further investigated the impacts of chronic stress on neutrophil‐associated brain injury in CAA. We found that CXCR2i treatment reversed the brain‐injurious effects of chronic stress as revealed by ameliorated Aβ40 deposition in brain blood vessels (Figure 2H), preserved ZO1 expression (Figure 2I), and attenuated white matter damage (Figure 2J) in both CXCR2i‐treated WT‐ and CAA‐CRS mice (Figure 2H–J). Our data illustrates that chronic stress exacerbates CAA outcomes through enhancing the destructive effects of neutrophil.

### 2.2 Neutrophil Extracellular Traps (NETs) Damage Brain Blood Vasculature which is Deteriorated During Chronic Stress

**Figure 3:**
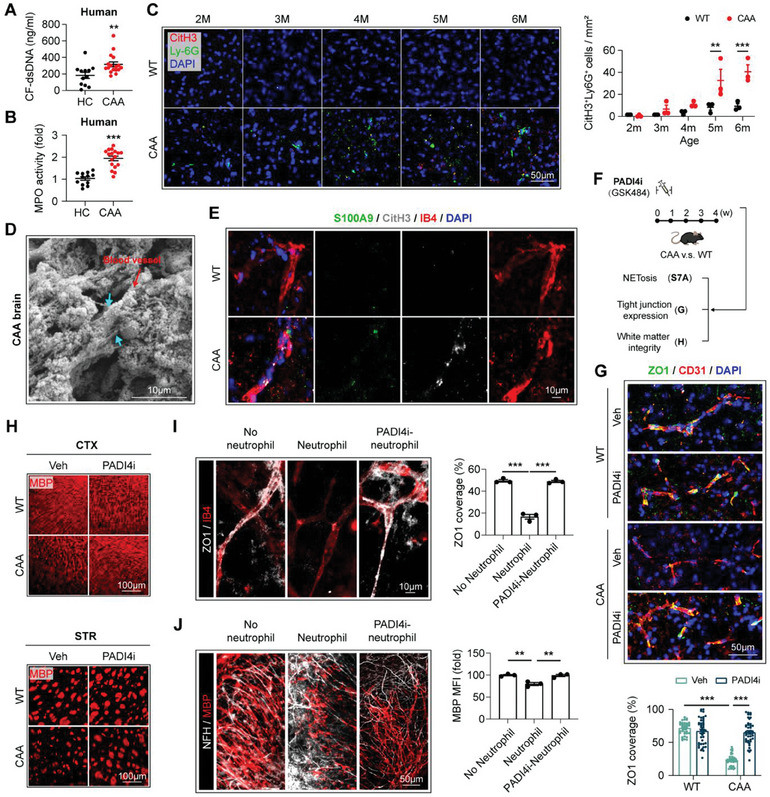
NETs accumulate along brain blood vessels in CAA. A,B) Plasma of the recruited CAA patients and age‐ and sex‐matched HC was subjected to analyses of cell free‐double strand DNA (CF‐dsDNA) A) and MPO activity B). *N* = 12 in HC group. *N* = 17 in CAA groups. ^**^ *p* < 0.01, ^***^ *p* < 0.001; by two‐tailed Student's *t* test. C) WT or CAA mice were sacrificed at indicated age and coronal brain sections were subjected to immunostaining of Ly6G and CitH3. Representative images showing NET accumulation in cortex are displayed. *N* = 3 for each time point. ^**^ *p* < 0.01, ^***^ *p* < 0.001; by two‐way *ANOVA*. D) Brain tissue of CAA mice (age = 32w, cortex) was subjected to scanning electron microscopy (SEM). Blue arrows emphasize NETs. The red arrow emphasizes blood vessel. E) Coronal brain sections of WT and CAA mice (age = 24w) were subjected to immunostaining of S100A9, CitH3, and IB4 and subsequent confocal microscopy. Representative images showing the accumulation of NETs (S100A9^+^CitH3^+^) along blood vessels (IB4^+^) in cortex are displayed. F–H) CAA or WT mice (age = 12w) were treated with PADI4i (GSK484, i.p., 4 mg k^−1^g, 1w) and sacrificed at 4w after treatment onset. An equal volume of PBS (vehicle, Veh) was injected to the control groups. NET‐inhibition efficacy of PADI4i treatment was validated with immunostaining of Ly6G and CitH3 (Figure S7A, Supporting Information). *N* = 3 mice in each group. F) Experimental design. G) Coronal brain sections were subjected to immunostaining of ZO1 and CD31. ZO1 coverage in 40–44 blood vessels in each group was analyzed respectively. ^***^ *p* < 0.001; by one‐way *ANOVA*. H) White matter integrity in cortex (CTX) and striatum (STR) was assessed with MBP staining. Statistic results are shown in Figure S7B (Supporting Information). I,J) Experimental design is shown in Figure S7C (Supporting Information). Briefly, OSCs were prepared with the cerebellum of CAA mice. BMDN were extracted from WT mice then pretreated with PADI4i (250 nm, 1 h, PADI4i‐neutrophil). Neutrophils with or without PADI4i pre‐treatment were then cocultured with the OSCs through transwell for 4 h (5 × 10^4^ BMDN per culture system with three brain slices). Experiments were repeated 3 times. I) ZO1 and IB4 were co‐stained to evaluate the tight junction loss of OSC after co‐cultured with BMDN. ^***^ *p* < 0.001; by one‐way *ANOVA*. J) White matter integrity was assessed with immunostaining of MBP and NFH. ^**^ *p* < 0.01; by one‐way *ANOVA*.

NET, which contains numerous ROS and tissue‐damaging enzymes including neutrophil elastase and myeloperoxidase (MPO), represents a destructive mediator derived from activated neutrophils.^[^ ^17^ ^]^ In recruited CAA patients, increased circulatory cell‐free double‐strand DNA (CF‐dsDNA) (**Figure** **3**A) and MPO activity (Figure 3B) were recorded, which are both main components of NETs. With immunostaining of the NET‐marker citrullinated histone 3 (CitH3), we documented NET formation in the brain of CAA mice (Figure 3C), which deteriorated along with age and coincided with the brain‐targeting chemotaxic dynamics of neutrophils (Figure 2A). As revealed by scanning electron microscopy (SEM) (Figure 3D) and immunostaining (Figure 3E), NETs in CAA brain mainly distributed along blood vessels, suggesting the possibility that the brain vascular damage in CAA could be attributed to NETs.

We thus studied the necessity of NETs in the neutrophil‐associated brain injury in CAA. We found that administration of the specific PADI4 inhibitor GSK484 (PADI4i, i.p., 4 mg k^−1^g, qd, for 7 days, Figure 3F), which prohibits NETs formation (Figure S7A, Supporting Information), ameliorated ZO1 loss (Figure 3G) and white matter injury (Figure 3H) in CAA mice. Consistently, pretreatment of PADI4i reversed the destructive effects of neutrophils to CAA OSCs (Figure 3I,J) as revealed by preserved ZO1 (Figure 3I) and MBP (Figure 3J) expression, while NETs formation in this system was verified (Figure S13E, Supporting Information). This approach is consistent with our hypothesis that NETs, once formed, release harmful substances such as myeloperoxidase (MPO) and neutrophil elastase (NE) that can damage the vascular wall and surrounding tissues. The results illustrate that NET is implicated in the injurious activities of neutrophils in CAA.

**Figure 4:**
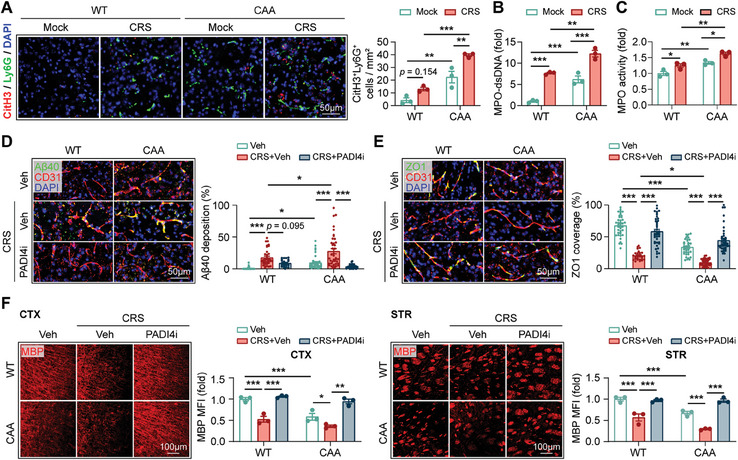
Suppressing NET formation protects against CAA in the context of chronic stress. A–C) CAA and WT mice (age = 12w) were stimulated with CRS for 4w. A) Coronal brain sections were subjected to immunostaining of Ly6G and CitH3. Representative confocal images showing the accumulation of NETs (Ly6G^+^CitH3^+^) in cortex are displayed. *N* = 3 in each group. ^**^ *p* < 0.01, ^***^ *p* < 0.001; by one‐way *ANOVA*. B) MPO bound double‐strand DNA (MPO‐dsDNA) in plasma of mice was quantified. *N* = 3 in each group. ^**^ *p* < 0.01, ^***^ *p* < 0.001; by one‐way *ANOVA*. C) MPO activity in brain of mice was measured. *N* = 3 in each group. ^*^ *p* < 0.05, ^**^ *p* < 0.01; by one‐way *ANOVA*. D–F) CAA and WT mice (age = 12w) were subjected to CRS for 4w with or without PADI4i treatment (GSK484 i.p., 4 mg k^−1^g, at the first week of CRS). An equal volume of PBS (vehicle, Veh) was injected to the control groups. The experimental design was displayed in Figure S7D (Supporting Information). *N* = 3 in each group. ^*^ *p* < 0.05, ^**^ *p* < 0.01, ^***^ *p* < 0.001; by one‐way *ANOVA*. D) Aβ40 deposition along blood vessels (CD31^+^) as assessed with immunostaining. Aβ40 coverage in 22–40 blood vessels in each group was analyzed respectively. E) Tight junction (ZO1^+^) expression along blood vessels (CD31^+^) as assessed with immunostaining. ZO1 coverage in 30–42 blood vessels in each group was analyzed respectively. F) White matter integrity in cortex (CTX) and striatum (STR) was assessed with MBP staining.

In CAA‐ and WT‐CRS mice, we observed pronounced NET accumulation in brain (**Figure** **4**A), up‐regulated MPO‐bound dsDNA level in blood (Figure 4B), and increased MPO activity in brain (Figure 4C) compared with the non‐stressed littermates (Figure 4A–C). Administration of PADI4i (Figure S7D, Supporting Information) reversed the destructive impacts of chronic stress in both CAA‐ and WT‐CRS mice, including Aβ40 deposition (Figure 4D), declined ZO1 expression (Figure 4E), myelin loss (Figure 4F) and cognitive decline (Figure S8, Supporting Information). We observed similar neutrophil infiltration (Figure S7E, Supporting Information), NET formation (Figure S7F,G, Supporting Information), and Aβ deposition (Figure S7H, Supporting Information) in CUMS model. Specific inhibition of neutrophils chemotaxis or NETosis in the central nervous system by cisterna magna injection of CXCR2i or PADI4i consistently ameliorated status of CAA mice (Figure S9, Supporting Information). Therefore, we demonstrate that chronic stress not only provokes brain‐targeting chemotaxis of neutrophils (Figure 1D–F) but also promotes NET formation (Figure 4), thus enhances the injurious impacts of neutrophils in CAA.

### 2.3 STAT6 Signaling Mediates the NET‐Inducing Effect of Aβ in CAA

**Figure 5:**
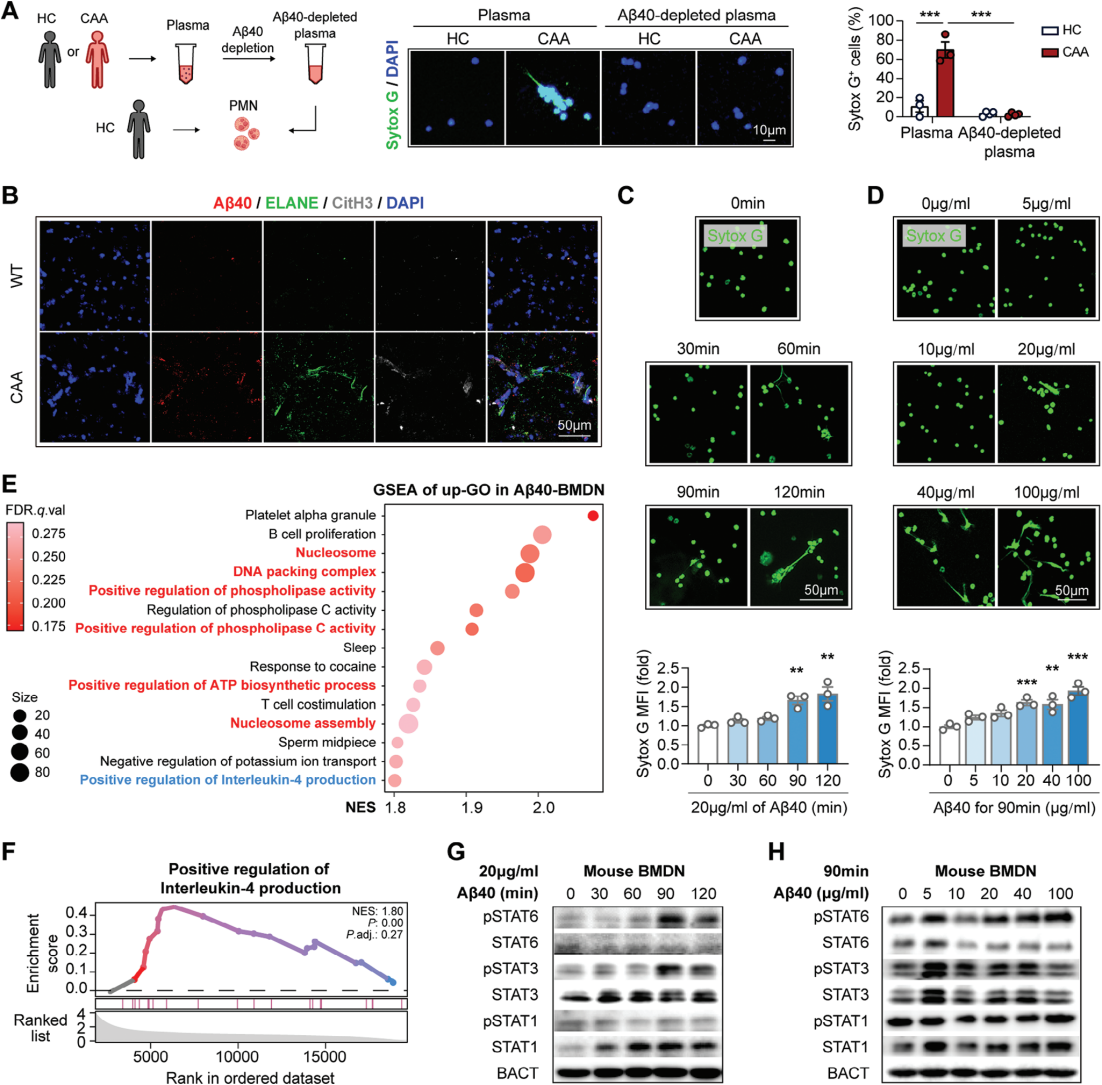
Aβ elicits NET formation through activating STAT6 signaling. A) Aβ40 in plasma of HC or CAA patients was pre‐depleted with anti‐Aβ40 antibodies‐bound beads. IgG‐bound beads were used as depletion control. Polymorphonuclear leukocytes (PMN) were isolated from peripheral blood of heathy donors. The DNA dye of Sytox green (Sytox G) was applied to reveal the extracellular DNA traps. Experiments were repeated for 3 times. B) Coronal brain sections of WT and CAA mice (age = 24w) were subjected to immunostaining of Aβ40, ELANE, and CitH3 then subsequent confocal microscopy. Representative images showing the colocalization of NETs (ELANE^+^CitH3^+^) and Aβ40 in cortex are displayed. C–H) BMDN were isolated from WT mice and treated with Aβ40. C,D) BMDN were treated with 20 µg mL^−1^ of Aβ40 for various time period C) or Aβ40 at various concentration for 90 min D). Sytox G was applied to reveal the extracellular DNA traps. Sytox G MFI was assessed with a plate reader and normalized to the non‐treated group. BMDN were then fixed and subjected to fluorescence microscopy. Experiments were repeated for 3 times. ^**^ *p* < 0.01, ^***^ *p* < 0.001; compared with 0 min C) or 0 µg mL^−1^ D) group; by one‐way *ANOVA*. E,F) BMDN with or without Aβ40 treatment (20 µg mL^−1^, 90 min) were subjected to bulk RNAseq. *N* = 2 in each group. GO of the up‐DEGs in Aβ40‐treated BMDN (Aβ40‐BMDN) was further subjected to GSEA. The up‐regulated nucleus‐modifying signaling has been highlighted (red) E). Plot showing the up‐regulation of “Positive regulation of Interleukin‐4 production” signaling in Aβ40‐BMDN is displayed F). G,H) BMDN were treated with 20 µg mL^−1^ of Aβ40 for various time period G) or Aβ40 at various concentration for 90 min H) then subjected to western blot. Experiments were repeated for 3 times. Statistic results are shown in Figure S10C,D (Supporting Information).

We next explored the molecular mechanisms of NET formation in CAA. Recent studies reveal that Aβ is an efficient NET inducer^[^ ^23^ ^]^ In consistence, we recorded that Aβ40, the key pathological molecule in CAA, was sufficient and necessary to induce NETs (**Figure** **5**A–D). In in vitro experiments, human neutrophils were isolated from the peripheral blood of healthy donors then stimulated with plasma of HC or CAA patients (Figure 5A). We found that CAA plasma efficiently elicited NET formation, while the effect was abolished when Aβ40 was pre‐depleted from plasma (Figure 5A). As revealed by immunostaining, NETs (ELANE^+^CitH3^+^) in CAA brains highly co‐localized with Aβ40 (Figure 5B), emphasizing the necessity of Aβ40 for NET formation in CAA. Moreover, we recorded that the NET‐inducing effect of Aβ40 strengthened along time (Figure 5C; Figure S10A,C, Supporting Information) and dose (Figure 5D, Figure S10A,D, Supporting Information). As assessed with bulk RNAseq, Aβ40‐treated BMDN displayed up‐regulated expression of nucleus‐modifying genes (Figure 5E), which further demonstrated the sufficiency for NET induction of Aβ40. In previous study, we have also found the detrimental metabolite from brains, including Aβ, have the potential to induce NETosis.^[^ ^20^ ^]^ These findings may explain the induction of NETs observed in our OSCs coculture experiments (Figures 2C,D and 3I,J). Meanwhile, WT OSCs exposed to Aβ40‐induced NETs demonstrated comparable disruptions in tight junctions and demyelination (Figure S13C,D, Supporting Information), underscoring the detrimental impact of Aβ40‐induced NETs on the integrity of the blood‐brain barrier.

To be noticed, the RNAseq data revealed that molecular signaling associated with IL‐4 production was enhanced in Aβ40‐treated BMDN (Figure 5E,F). Members of STAT family are vital regulators of IL‐4 productivity.^[^ ^24^ ^]^ As assessed with western blot (Figure 5G,H; Figure S10C,D, Supporting Information) and flow cytometry (Figure S10E, Supporting Information), we found that it was STAT6 signaling that was activated in BMDN by Aβ40. On the other hand, we did not record pronounced alteration of inflammatory property in the Aβ40‐treated BMDN (Figure S10F, Supporting Information).

**Figure 6:**
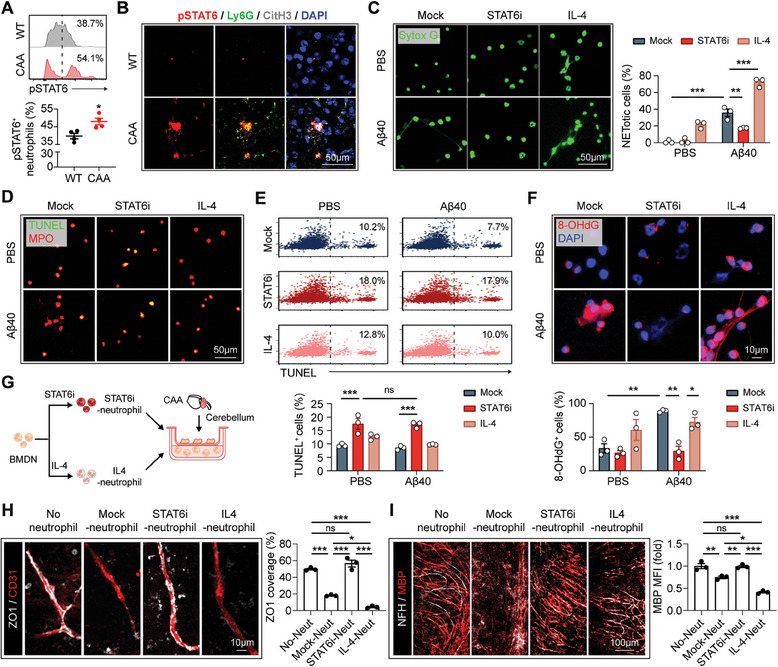
STAT6 is the switch between apoptosis and NETosis. A) Brain cells of CAA or WT mice (age = 16w) were subjected to flow cytometric analysis. Expression of pSTAT6 (Tyr641) in neutrophils was calculated. *N* = 4 in each group. B) Coronal brain sections of WT and CAA mice (age = 24w) were subjected to immunostaining of pSTAT6 (Tyr641), Ly6G and CitH3 and subsequent confocal microscopy. Representative images showing STAT6 activation (pSTAT6^+^) in NET‐forming (CitH3^+^) neutrophils (Ly6G^+^) in cortex are displayed. C–F) BMDN isolated from WT mice were pre‐treated with or without STAT6i (1 µm) or IL‐4 (20 ng mL^−1^) for 1 h, then stimulated with Aβ40 (20 µg mL^−1^) for 90 min. ^*^ *p* < 0.05, ^**^ *p* < 0.01, ^***^ *p* < 0.001; by one‐way *ANOVA*. C) Sytox G was applied to reveal the extracellular DNA traps. Experiments were repeated for 3–4 times. D,E) Apoptosis of BMDN was assessed with TUNEL staining and subsequent microscopy D) or flow cytometric analysis E). Experiments were repeated for 3–4 times. F) Intracellular ROS accumulation was evaluated with 8‐OHdG staining. Experiments were repeated 3 times. G–I) OSCs were prepared with the cerebellum of CAA mice. BMDN were extracted from WT mice then pretreated with STAT6i (1 µm, STAT6i‐neutrophil) or IL‐4 (20 ng mL^−1^, IL4‐neutrophil) for 1 h. BMDN were then cocultured with the OSCs through transwell for 4 h (5 × 10^4^ BMDN per culture system with three brain slices). Experiments were repeated for 3 times. G) Experimental design. (H) ZO1 and CD31 were co‐stained to evaluate the tight junction loss of OSCs after co‐cultured with BMDN. ^*^ *p* < 0.05, ^***^ *p* < 0.001; by one‐way *ANOVA*. I) White matter integrity was assessed with immunostaining of MBP and NFH. ^*^ *p* < 0.05, ^**^ *p* < 0.01, ^***^ *p* < 0.001; by one‐way *ANOVA*.

We further evaluated the activity of STAT6 signaling in vivo. With flow cytometric analysis (**Figure** **6**A) and immunostaining (Figure 6B), we found that the expression of phospho‐STAT6 (pSTAT6, Tyr641) was increased in neutrophils in CAA brains. To be noticed, the pSTAT6^+^ neutrophils in CAA brains displayed pronounced CitH3 expression (Figure 6B). In vitro, we found that suppressing STAT6 signaling with the specific inhibitor of AS1517499 (STAT6i, 1 µm, 1 h, pretreatment) reversed Aβ40‐induced NET formation (Figure 6C), suggesting that STAT6 activation is indispensable for the NET‐inducing effect of Aβ40.

It is established that STAT6 activation prevents the programmed cell death of apoptosis.^[^ ^25^ ^]^ Indeed, to enhance STAT6 activity with either Aβ40 or IL‐4 forbade neutrophil apoptosis as revealed by TUNEL analysis (Figure 6D,E). However, intracellular ROS increased (Figure 6F) and NET was elicited (Figure 6C) upon STAT6 activation. Consequently, the tissue‐destructing effects of neutrophils were amplified (Figure 6G–I; Figure S13A,B, Supporting Information). By contrast, treatment of STAT6i induced apoptosis in neutrophils (Figure 6D,E) but prevented intracellular ROS accumulation (Figure 6F) and NET formation (Figure 6C). Accordingly, neutrophils pretreated with STAT6i (STAT6i‐neutrophil) were innocuous to CAA OSCs (Figure 6G–I). These data indicate that STAT6 is the switch between apoptosis and NETosis. Aβ40 activates STAT6 signaling thus directs programmed neutrophil death from apoptosis toward NETosis.

### 2.4 Chronic Stress Promotes NET Formation by Enhancing STAT6 Activation Through Norepinephrine

**Figure 7:**
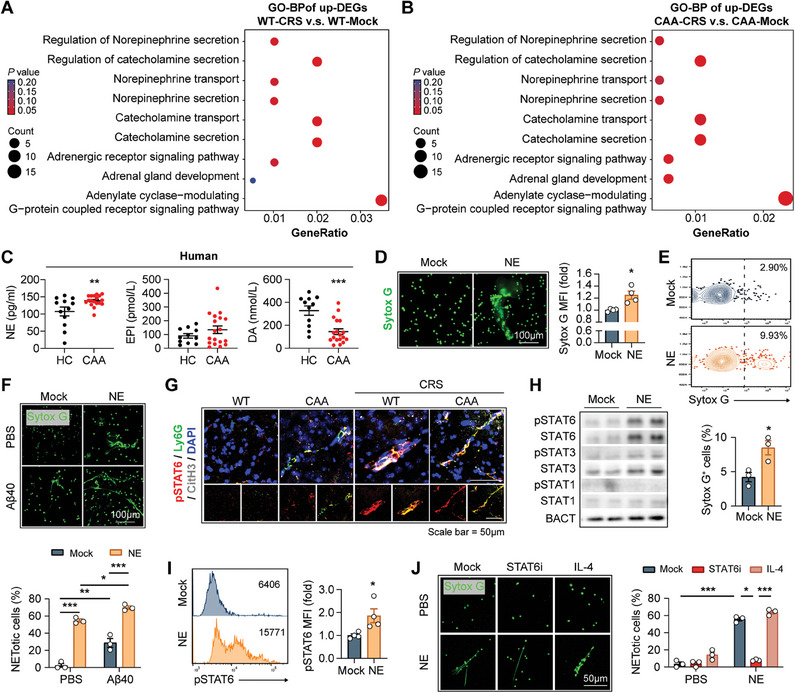
Norepinephrine exacerbates NET formation in CAA in the context of chronic stress. A,B) Brain tissue of WT or CAA mice with or without CRS stimulation was subjected to bulk RNAseq. *N* = 2 mice in each group. GO‐BP analysis of the up‐regulated DEGs in WT‐CRS A) or CAA‐CRS B) mice compared to the non‐stimulated controls (WT‐Mock or CAA‐Mock) was performed. C) Concentration of Norepinephrine (NE), Epinephrine (EPI), and Dopamine (DA) in plasma of the recruited CAA patients and HC was calculated. *N* = 11 in HC group. *N* = 18 in CAA groups. ^**^ *p* < 0.01, ^***^ *p* < 0.001; by Student's *t* test. D,E) BMDN were isolated from WT mice and treated with NE (0.1 µm, 2 h). Sytox G was applied to reveal the extracellular DNA traps. D) Sytox G MFI was assessed with a plate reader and normalized to the non‐treated group. BMDN were then fixed and subjected to fluorescence microscopy. Experiments were repeated for 4 times. E) The percentage of Sytox G^+^ BMDN was calculated with flow cytometry. Experiments were repeated for 3 times. F) BMDN were isolated from WT mice and treated with NE (0.1 µm) with or without Aβ40 (20 µg mL^−1^) for 2 h. Sytox G was applied to reveal the extracellular DNA traps. G) Coronal brain sections of WT and CAA mice with or without CRS stimulation (age = 16w) were subjected to immunostaining of pSTAT6 (Tyr641), Ly6G, and CitH3 and subsequent confocal microscopy. Representative images showing STAT6 activation (pSTAT6^+^) in NET‐forming (CitH3^+^) neutrophils (Ly6G^+^) in cortex are displayed. H,I) BMDN were isolated from WT mice and treated with NE (0.1 µm, 2 h). Western blot H) and flow cytometric analysis I) were performed to evaluate STAT6 activation in BMDN. Experiments were repeated for 3–4 times. Statistic results of the western blot are shown in Figure S14C (Supporting Information). J) BMDN isolated from WT mice were pre‐treated with or without STAT6i (1 µm) or IL‐4 (20 ng mL^−1^) for 1 h, then treated with NE (0.1 µm) for 2 h. Sytox G was applied to reveal the extracellular DNA traps. Experiments were repeated 3 times. ^*^ *p* < 0.05, ^**^ *p* < 0.01; by one‐way *ANOVA*.

We went on to study how chronic stress exacerbated NET formation and the consequent brain injury in CAA. With GO analysis, we found that the expression of catecholamine and downstream signaling‐associated genes was up‐regulated in both WT‐ (**Figure** **7**A) and CAA‐CRS models (Figure 7B). Considering the pathological roles of catecholamines in psychological disorders, we speculated that catecholamines were associated with the detrimental impacts of chronic stress on CAA outcomes. Therefore, circulatory Norepinephrine (NE), Epinephrine (EPI), and Dopamine (DA) in the recruited cohort were measured and we found that the plasma NE was increased in CAA patients compared with HC (Figure 7C). Meanwhile, the level of NE in plasma of mice received chronic stress also elevated (Figure S14E,F, Supporting Information). Notably, we observed that NE efficiently induced NET formation (Figure 7D,E; Figure S14A,B, Supporting Information), which was even more prominent in the presence of Aβ40 (Figure 7F). Moreover, we found that pSTAT6 expression was up‐regulated in neutrophils in CAA‐CRS brains in vivo (Figure 7G) and NE‐treated BMDN in vitro (Figure 7H,I; Figure S14C, Supporting Information), and expression of β‐AR upregulated on neutrophils in brains of mice received chronic stress (Figure S14D, Supporting Information). NE‐dependent NET formation was suppressed by STAT6i while was deteriorated by IL‐4 treatment (Figure 7J), indicating that STAT6 mediated the NET‐inducing effect of NE. The data illustrates that chronic stress promotes NET formation and the subsequent brain injury in CAA by enhancing STAT6 activation in neutrophil through NE. We thus propose that chronic stress‐associated psychological disorders and NETs are both promising therapeutic targets in CAA.

## 3 Discussion

The current study explores the impacts of chronic stress on CAA progression. We report that Aβ activates STAT6 signaling in neutrophils, which prevents apoptosis and switches the programmed cell death toward NETosis. Chronic stress provokes neutrophil chemotaxis towards brain vasculature, and further enhances STAT6 activation in neutrophil through NE, thus exacerbates NET formation and consequent brain injury. We propose that tackling chronic stress‐associated psychological disorders and inhibiting NET formation are potential therapeutic strategies against CAA.

In our study, the observation that invasive neutrophils in the CAA brain release neutrophil extracellular traps (NETs) along blood vessels, exacerbating Aβ deposition and subsequent brain injury, is alongside the finding that Aβ itself activates STAT6 signaling in neutrophils to induce NET formation. This initially appears to create a paradox in the causality of the pathological mechanisms at play in CAA. As far as we are concerned, it is plausible that these observations are part of a sophisticated feedback loop that drives the disease process forward. The initial accumulation of Aβ in the cerebral vasculature could serve as a potent activator of neutrophils, leading to the formation of NETs, which are known to contain a variety of pro‐inflammatory and pro‐thrombotic molecules. These NETs, once released into the extracellular environment, might not only directly exacerbate endothelial damage and inflammatory responses but also create a scaffold that further promotes Aβ aggregation and deposition around the blood vessels. This escalation in Aβ deposition could then result in further recruitment and activation of neutrophils, perpetuating the cycle. Each iteration of this loop could amplify the pathological environment within the brain, leading to increasingly severe vascular and neuronal damage. This model aligns with existing literature on the role of chronic inflammation and immune cell activation in CAA.^[^ ^3^, ^23^, ^26^ ^]^

In spite of the short lifespan, neutrophils are implicated in the pathophysiology of numerous chronic diseases including rheumatoid arthritis^[^ ^27^ ^]^ and systemic lupus erythematosus (SLE).^[^ ^28^ ^]^ The current study unveils the detrimental roles of neutrophils in CAA development. The invasive neutrophils in CAA brain release NETs along blood vessels, which exacerbates Aβ deposition and brain injury. Therefore, neutrophil is a potential therapeutic cell target against brain amyloid angiopathy in CAA.

Neuropsychological symptoms in CAA patients are generally considered as the consequences of CAA pathology. Indeed, brain injury could result in psychological disorders. For example, locus coeruleus (LC), the major noradrenergic nerve nucleus in brain, degenerates in AD,^[^ ^29^, ^30^ ^]^ which leads to mental disorders such as anxiety and depression.^[^ ^31^ ^]^ Nevertheless, contributions of chronic stress to psychological abnormality could not be neglected. Chronic stress is implicated in the development of organic diseases including cancer^[^ ^9^, ^32^, ^33^ ^]^ and cardiovascular disorders.^[^ ^34^ ^]^ In the current study, we report that chronic stress exacerbates CAA outcomes, suggesting that it is a stimulus of CAA progression. Therefore, stress‐associated psychological disorders could be more than just consequences of CAA. Chronic stress induces elevated level of Norepinephrine in the circulation, which can also trigger NETs formation, along with the peripheral deposition of Aβ,^[^ ^35^, ^36^ ^]^ and supports the hypothesis that peripheral NETs may have a key impact on the disruption of the blood‐brain barrier in CAA. However, the cerebral vasculature in CAA bears the brunt of Aβ deposition, where neutrophil adhesion and local NETs formation play an indispensable role in the pathogenesis of CAA. Moreover, stress‐stimulated WT mice displayed Aβ accumulation in brain vasculature, indicating that chronic stress could initiate amyloid angiopathy. Therefore, mutual casualty and ‐promotion between psychological abnormality and CAA pathophysiology may exist.

We have observed that chronic stress promotes neutrophil chemotaxis towards brain. To be noticed, the brain‐targeting neutrophils appear to retend within blood vessels rather than penetrate into brain parenchyma. According to previous reports, chronic stress could cause brain blood vascular dysfunction.^[^ ^37^ ^]^ Therefore, we speculate that the intra‐vessel distribution of brain‐invasive neutrophils in response to chronic stress could be attributed to the increment of adhesion molecules, chemokines, or other neutrophil attractants in blood vessel cells. On the other hand, in CAA models without stress stimulation, neutrophils in brain are restricted within blood vessels at early age (3–4 months) probably due to the intra‐vessel accumulation of Aβ. In CAA mice, locally condensed Aβ in the brain is sufficient to induce NETosis, and these Aβ‐induced NETs effectively disrupt vasculature. Nevertheless, neutrophils intrude into brain parenchyma as CAA progresses, which could be the result of BBB breakdown in developed CAA.

In the current study, we report that chronic stress leads to increased NET formation by activating STAT6 through NE. However, the functions of adrenergic receptors (AR) in neutrophils are under debate. One study suggests that adrenaline reduces stimulus‐dependent cell activation of human neutrophils by activating

β2‐AR,^[^ ^38^ ^]^ while another report has shown that β1‐AR blockade suppresses neutrophil activation thus reduces the brain‐damaging effects.^[^ ^39^ ^]^ In acute phase of inflammation, β2‐AR acts as co‐activator to enhance TLR pathway's response to LPS in innate immune cells and promote IL‐10 autosecretion to further modulate the inflammatory response,^[^ ^40^ ^]^ while during the long‐term reparative phase, chronic activation of β2‐AR on myeloid cells is implicated to fibrotic process following myocardial infarction (MI) with up‐regulation of *Anax1*.^[^ ^41^, ^42^ ^]^ These findings illustrate that various functional impacts of AR in neutrophils exist, which could be dependent on the subtype, co‐activators, and intro‐cellular coordinators of the receptors.

The origin of brain‐invasive neutrophils in CAA remains to be studied. Meningeal lymphatic vessel is regarded as a path for leukocyte infiltration in multiple sclerosis.^[^ ^43^ ^]^ In the context of CAA, we observed elevated neutrophil count in peripheral blood of both patients and mouse models. Therefore, we speculate that neutrophils in CAA brains are at least partially originated from blood. Circulatory NE is mainly derived from adrenal gland and noradrenergic peripheral nerve. We have documented that plasma NE level is increased in CAA patients, which supports that neutrophils are activated in blood during chronic stress then subsequently target towards brain.

In conclusion, the current study elucidates that chronic stress is a deteriorating factor of CAA, which provokes neutrophil chemotaxis towards brain vasculature and induces NET formation. Chronic stress‐associated psychological disorders, neutrophils, and NETs are potential therapeutic targets in CAA.

## 4 Experimental Section

### Animals

All animal experimental studies were approved by the Animal Care and Use Committee of Sun Yat‐Sen University, and performed based on ARRIVE guidelines. Healthy wild‐type C57/BI6 mice (8‐ to 24‐week‐old) and the age‐and‐gender‐matched homozygous transgenic Swedish Dutch Iowa (Tg‐SwDI^+/+^) mice with C57/Bl6 background generated with knocked in of human amyloid‐β precursor protein (APP) gene with three familial mutations, utilized as CAA murine models, were obtained from Guangdong Medical Laboratory Animal Center (Guangzhou, China), and housed in humidity‐ and temperature‐controlled barrier cages in Sun Yat‐sen University with a 12‐h light‐dark cycle. Food and water were sufficient and freely accessible.

### Chronic Stress Model

Twelve‐week‐old mice were restrained in 50 mL conical tubes to prevent them from moving freely or turning around for 2 h per day for 28 consecutive days. In experiments with neutrophil infiltration inhibitory treatment, SB225002 (MCE) was dissolved in corn oil and administered to mice (2 mg k^−1^g, i.p., qd. for 3w). In experiments with PADI4i treatment, GSK484 (MCE) was dissolved in corn oil and administered to mice (4 mg k^−1^g, i.p., qd. for 1w).

### Chronic Unpredictable Mild Stress Model

Eight‐week‐old male mice were subjected to two or three stressors per day, for 28 consecutive days. The stressors were selected randomly among the following list: tail pinch (5 min, 1 cm from the distal portion of the tail); physical restraint, cold swimming (5 min at 4 °C); food deprivation (24 h); water deprivation (24 h); moist bedding (24 h); removal of all bedding (24 h); 30° cage tilt (3 h); overnight illumination. All mice from the CUMS group (n = 6) received the same combination of stressors every day. No individual stressor was repeated on two consecutive days.

### Primary Mouse Bone Marrow‐Derived Neutrophil Culture and Treatment

Neutrophils were isolated from bone marrow of 8‐week‐old male mice by Percoll (GE Healthcare) gradient centrifugation according to published methodology^[^ ^44^ ^]^ and suspended in RPMI‐1640 medium (eLGbio) containing 10% FBS and 1% penicillin‐ streptomycin (PS). BMDN were incubated at 37 °C and 5% CO_2_ and treated with PADI4i (GSK484, 250 nM, MCE), STAT6i (AS15179, 1 µm, MCE) and IL‐4 (20 ng mL^−1^, Peprotech) for 1 h, Norepinephrine (0.1 µm, Macklin), Epinephrine (0.1 µm, Selleck) or Dopamine (0.1 µm, MCE) for 2 h in indicated experiments. In experiments with Sytox G staining, BMDN were incubated with Sytox green (25 nM, ThermoFisher) for 15 min. Aβ40‐NETs were generated and collected according to published research.^[^ ^45^ ^]^ Briefly. 0.1 million BMDN were exposed to 20 µg mL^−1^ Aβ40 for 4 h. After removal of the supernatant, NETs adhered at the bottom were washed down by pipetting 1 mL of pre‐cold PBS and were centrifuged at 1000 g at 4 °C for 10 min to collect the cell‐free but NETs‐abundant supernatant. The DNA concentration of NETs was measured by spectrophotometry and the NETs were used for further experiments.

### Immunofluorescence Staining

In in vivo experiments, mice were sacrificed at indicated time points. After sufficient perfusion with PBS then 4% paraformaldehyde, brains were removed and cut into coronal sections (25 µm) on a frozen microtome. In in vitro experiments, neutrophils were seeded on coverslips coated with poly‐L‐lysine (Sigma). After treatment, cells were fixed with 4% paraformaldehyde for 20 min. Brain sections or fixed BMDM were permeabilized with PBS containing 0.25% Triton‐X100 in PBS then blocked with 5% mouse serum, and incubated with primary antibodies overnight in PBS containing 0.03% Triton‐X100 and 3% BSA, then secondary antibodies for 1 h at room temperature. Antibodies used in immunofluorescence staining were indicated in Extended methods in Su pplementary Material. The coverslips or slices were finally mounted with anti‐fade fluorescence medium containing DAPI (Abcam ab104139) or not (Abcam ab104135). Images were captured with a confocal microscopy (Leica TSC SP8) and processed with NIH Image J software by a blinded observer for the unbiased counting of automatically recognized cells and mean fluorescent intensity calculation. For Neutrophils and NETs quantification, Ly6G^+^neutrophils and Ly6G^+^CitH3^+^NETs were counted in four fields per section under 20x objects of the second section, and results were averaged to generate the value for a single mouse. For MBP MFI qualification, cortex and striatum of the second section were imaged under 10x objects with the same parameters and analyzed with ImageJ without adjusting brightness and contrast.

### MPO Activity

Plasma MPO activity was measured with EL‐TMB Chromogenic Reagent kit (Sangon Biotech, C520026‐0500) under instructions of manufacturers.

### Organotypic Slice Culture

Organotypic cerebellar slice was obtained from P10 mouse pups according to published methodology.^[^ ^46^ ^]^ Briefly, after decapitation, the cerebellum was isolated in ice‐cold Hank's Balanced Salt Solution (HBSS) containing 1% penicillin‐streptomycin (PS). The isolated cerebellum was cut into 350 µm thick slices using a tissue chopper and was transferred to 0.4 µm culture plate inserts. Each culture insert contained three slices and was placed in twelve‐well plates containing 500 µL of culture medium per well. Culture medium was consisted of 0.5x minimum essential medium (MEM) containing 25% heat‐inactivated horse serum, 25% HBSS, 2 mm glutamine and 0.5% glucose. The slice cultures were kept at 37 °C in a humidified atmosphere (5% CO_2_) and the culture medium was refreshed every 2 days.

### Enzyme‐Linked Immunosorbent Assay (ELISA)

Plasma CF‐dsDNA was detected using the PicoGreen dsDNA reagent (Invitrogen) according to the manufacturer's instructions. MPO‐dsDNA was detected using a previously described ELISA method.^[^ ^45^ ^]^ Ninety‐six‐well black plated were coated with 7 µg mL^−1^ anti‐MPO polyclonal antibody (proteintech, 22225‐1‐AP‐100UL) overnight at 4 °C. After blocking in 1%BSA, plasma was added and incubated at 37 °C for 1 h. The concentration MPO‐bound dsDNA was then measured using the PicoGreen dsDNA reagent (Invitrogen). The concentration of NE, EPI, and DA in human plasma was measured with human NE ELISA kit (Dogesce, DG10156H), EPI ELISA kit (Dogesce, DG10980H), and DA ELISA kit (Dogesce, DG11811H) respectively. The concentration of NE, GC in mouse plasma were measured with mouse NE ELISA kit (Dogesce, DG30367M), GC ELISA kit (Dogesce, DG30281M) respectively. For Aβ40 measurement, mice brains were minced in pre‐cold 0.01 m PBS and homogenized, then centrifuged at 1000 g, 15 min, and supernatant were subjected to measured concentration of Aβ40 with mouse Aβ40 ELISA kit (MEIMIAN, MM‐0461M2).

### Scanning Electron Microscopy (SEM)

Cortex was cut and harvested using sharp blade quickly and washed with PBS gently, then immediately fixed by electron microscopy fixative (Servicebio, G1102) for 2 h at room temperature, then postfixed in 1% OsO4 in 0.1 m PB (pH 7.4) for 2 h, dehydrated with a graded ethanol series (25–100%), and critical point dried from CO_2_. The dried tissue was attached on metallic stubs with carbon stickers and sputter‐coated with gold for 30s. The sample was observed and imaged in a HITACHI scanning electron microscope at 5 kV.

### Flow Cytometric Analysis

In in vivo experiments, mice were perfused with PBS. Brains were isolated and subjected to digestion with 0.25% trypsin‐EDTA (ThermoFisher) at 37 °C for 20 min. Brain tissue was then pressed through a 70 µm cell strainer. With a density gradient centrifugation in 30%/70% Percoll solution, myelin and debris were removed. Brain cells were then washed with PBS and subjected to surface labeling. After being washed with PBS, cells were fixed and permeabilized (Invitrogen, Intracellular Fixation, and Permeabilization Buffer Set), then stained with intracellular antibodies. Peripheral blood of mice was collected with Heparin‐based anticoagulation. For isolation of bone marrow, femurs and tibias were isolated and cleaned. Bone lumen was exposed. Bone marrow was flushed with PBS using a 10 mL syringe with a 25G5/8 needle. Spleens were collected and disrupted through a wire grid with PBS. After filtration through a 70 µm cell strainer, spleen, and bone marrow samples were next centrifuged at 500 g for 5 min at 4 °C. Red blood cells were removed with ACK lysis buffer. Leukocytes were washed with PBS and subjected to surface labeling. Antibodies used in FACS were indicated in Expanded methods in Supporting Information. Apoptosis of BMDN was assessed using One Step TUNEL Apoptosis Assay Kit (Beyotime, C1088) according to instructions of manufacturers. Flow cytometric analysis was performed using a FACS flow cytometer (BD Biosciences, San Jose, CA), and data analysis was performed using FlowJo software (FlowJo, version 10.0, Ashland, OR).

### Western Blot

Protein was extracted with RIPA lysis buffer (Sigma) from cells. A total amount of 40 µg protein of each sample was applied to western blot experiments. Western blot was performed with standard SDS‐polyacrylamide gel electrophoresis method and ECL western HRP substrate (Affinity Biosciences KF8001‐500). Antibodies used in western blot were indicated in Expanded methods in Supporting Information. Immunoreactivity was assessed with Image J (NIH).

### Study Approval

The clinical experimental studies were approved by the Medical Ethics Committee of the Third Affiliated Hospital of Sun Yat‐Sen University. All participants had been given informed consent according to the principles illustrated in the Declaration of Helsinki.

### Study Population

A cohort consisting of 26 patients with CAA in neurology clinics and 24 age‐ and sex‐matched healthy controls in the medical examination department in the Third Affiliated Hospital of Sun Yat‐Sen University from July 2018 to June 2023 was recruited consecutively. All CAA patients recruited in the study were eligible for the inclusion criteria below:^[^ ^47^ ^]^ 1) Age ≥ 55y; 2) MRI or CT neuroimaging showing that multiple hemorrhages (ICH, CMB) restricted to lobar, cortical, or cortical‐subcortical regions (cerebellar hemorrhage allowed), or single lobar, cortical, or cortical‐subcortical hemorrhage and cortical superficial siderosis (focal or disseminated); 3) Absence of other cause of hemorrhage (differential diagnosis of lobar hemorrhages) including antecedent head trauma, hemorrhagic transformation of an ischemic stroke, arteriovenous malformation, hemorrhagic tumor, warfarin therapy with international normalization ratio > 3, vasculitis. To preclude the interference of vascular amyloid deposition to the impact of brain parenchymal amyloid deposition, AD patients with MRI or CT neuroimaging clues for CAA were precluded.

### Statistical Analysis

Test of normality was performed before the parametric analysis of Student's *t*‐test, one‐way *ANOVA*, and two‐way *ANOVA*. GraphPad Prism software (version 9.0) was used for statistical analysis.

### Statistical Analysis—Student's *t* Test

Unpaired parametric *t*‐test (two‐tailed) was performed in data comparison of two groups. Error bar represents the standard error of mean (SEM).

### Statistical Analysis—One‐way *ANOVA*

No matching or pairing *ANOVA* was performed in data comparison of three groups or more. The result was corrected for multiple comparisons using statistical hypothesis testing (Dunnett). The error bar represents SEM.

### Statistical Analysis—Two‐Way *ANOVA*

Two‐way *ANOVA* was performed in data on a quantitative dependent variable multiple levels of two categorical independent variables.

### Statistical Analysis—Spearman Correlation

The correlation between SAS and NLR of CAA patients was computed with nonparametric Spearman correlation coefficients.

## Conflict of Interest

The authors declare no conflict of interest.

## Author Contributions

H.H. and X.D. contributed equally to the work. H.H. designed and performed the experiments, collected and analyzed the data, and drafted the manuscript. X.D. contributed to the experimental design and revised the manuscript. C.L. and S.W. contributed to the experimental design and the manuscript. Y.W. and S.L. collected the clinical samples. T.L. collected the clinical data and revised the manuscript. S.S. and M.H. performed the animal experiments and collected the data. Z.L. and W.C. designed and supervised the study and critically revised the manuscript. The authors read and approved the final manuscript.

## Ethics Approval

All animal experiments were approved by the Third Affiliated Hospital of Sun Yat‐sen University and performed following the Guide for the Care and Use of Laboratory Animals and Stroke Treatment. Clinic research was approved by the ethics committee of the Third Affiliated Hospital of Sun Yat‐sen University.

## Supporting information



Supporting Information

## Data Availability

The data that support the findings of this study are available from the corresponding author upon reasonable request.

## References

[1] M. Hu , T. Li , X. Ma , S. Liu , C. Li , Z. Huang , Y. Lin , R. Wu , S. Wang , D. Lu , T. Lu , X. Men , S. Shen , H. Huang , Y. Liu , K. Song , B. Jian , Y. Jiang , W. Qiu , Q. Liu , Z. Lu , W. Cai , Nat. Commun. 2023, 14, 3945. 37402721 10.1038/s41467-023-39693-x PMC10319857

[2] C. V. DeSimone , J. Graff‐Radford , M. A. El‐Harasis , A. A. Rabinstein , S. J. Asirvatham , D. R. Holmes Jr. , J. Am. Coll. Cardiol. 2017, 70, 1173. 28838368 10.1016/j.jacc.2017.07.724

[3] E. E. Smith , S. Crites , M. Wang , A. Charlton , A. Zwiers , R. Sekhon , T. Sajobi , R. Camicioli , C. R. McCreary , R. Frayne , Z. Ismail , J. Am. Heart Assoc. 2021, 10, e022089. 34755541 10.1161/JAHA.121.022089 PMC8751932

[4] M.‐T. Shindo , Front. Aging Neurosci. 2023, 15, 1143834. 37032819 10.3389/fnagi.2023.1143834 PMC10079999

[5] N. F. Case , A. Charlton , A. Zwiers , S. Batool , C. R. McCreary , D. B. Hogan , Z. Ismail , C. Zerna , S. B. Coutts , R. Frayne , B. Goodyear , A. Haffenden , E. E. Smith , Stroke 2016, 47, 2010. 27338926 10.1161/STROKEAHA.116.012999

[6] M. Planton , N. Raposo , J. F. Albucher , J. Pariente , Rev. Neurol. 2017, 173, 562. 28993004 10.1016/j.neurol.2017.09.006

[7] S. Chiba , T. Numakawa , M. Ninomiya , M. C. Richards , C. Wakabayashi , H. Kunugi , Prog. Neuropsychopharmacol. Biol. Psychiatry 2012, 39, 112. 22664354 10.1016/j.pnpbp.2012.05.018

[8] M. E. A. Tschaffon‐Müller , E. Kempter , L. Steppe , S. Kupfer , M. R. Kuhn , F. Gebhard , C. Pankratz , M. Kalbitz , K. Schütze , H. Gündel , N. Kaleck , G. Strauß , J. Vacher , H. Ichinose , K. Weimer , A. Ignatius , M. Haffner‐Luntzer , S. O. Reber , Nat. Commun. 2023, 14, 3262. 37277336 10.1038/s41467-023-38616-0 PMC10241819

[9] B. Cui , Y. Luo , P. Tian , F. Peng , J. Lu , Y. Yang , Q. Su , B. Liu , J. Yu , X.i Luo , L. Yin , W. Cheng , F. An , B. He , D. Liang , S. Wu , P. Chu , L. Song , X. Liu , H. Luo , J. Xu , Y. Pan , Y. Wang , D. Li , P. Huang , Q. Yang , L. Zhang , B. P. Zhou , S. Liu , G. Xu , et al., J. Clin. Invest. 2019, 129, 1030. 30688660 10.1172/JCI121685 PMC6391112

[10] M. Kivimaki , A. Steptoe , Nat. Rev. Cardiol. 2018, 15, 215. 29213140 10.1038/nrcardio.2017.189

[11] Q. Deng , H. Chen , Y. Liu , F. Xiao , L. Guo , D. Liu , X. Cheng , M. Zhao , X. Wang , S. Xie , S. Qi , Z. Yin , J. Gao , X. Chen , J. Wang , N. Guo , Y. Ma , M. Shi , Brain Behav. Immun. 2016, 57, 243. 27133786 10.1016/j.bbi.2016.04.017

[12] L. Duan , J. Chen , M. Razavi , Y. Wei , Y. Tao , X. Rao , J. Zhong , Front. Immunol. 2019, 10, 501. 30941135 10.3389/fimmu.2019.00501 PMC6433825

[13] Q. Li , J. Zhang , Z. Gao , Y. Zhang , J. Gu , Microb. Pathog. 2023, 176, 106008. 36736544 10.1016/j.micpath.2023.106008

[14] Y. Ishikawa , T. Furuyashiki , Neurosci. Res. 2022, 175, 16. 34606943 10.1016/j.neures.2021.09.005

[15] S. Foertsch , S. O. Reber , Neurosci. Biobehav. Rev. 2020, 113, 169. 32109454 10.1016/j.neubiorev.2020.02.025

[16] F. V. S. Castanheira , P. Kubes , Blood 2019, 133, 2178. 30898862 10.1182/blood-2018-11-844530

[17] V. Papayannopoulos , Nat. Rev. Immunol. 2018, 18, 134. 28990587 10.1038/nri.2017.105

[18] H. Block , J. Rossaint , A. Zarbock , Cells 2022, 11, 1919. 35741047 10.3390/cells11121919 PMC9222025

[19] A. Warnatsch , M. Ioannou , Q. Wang , V. Papayannopoulos , Science 2015, 349, 316. 26185250 10.1126/science.aaa8064 PMC4854322

[20] W. Cai , S. Liu , M. Hu , F. Huang , Q. Zhu , W. Qiu , X. Hu , J. Colello , S. G. Zheng , Z. Lu , Transl. Stroke Res. 2020, 11, 108. 30847778 10.1007/s12975-019-00694-y PMC6993940

[21] X. Wu , Y. Yang , Int. Immunopharmacol. 2024, 133, 112085. 38626550 10.1016/j.intimp.2024.112085

[22] N. A. Tan , A. M. A. Carpio , H. C. Heller , E. C. Pittaras , Genes (Basel) 2023, 15, 47. 38254938 10.3390/genes15010047 PMC10815655

[23] H. Munir , J. O. Jones , T. Janowitz , M. Hoffmann , M. Euler , C. P. Martins , S. J. Welsh , J. D. Shields , Nat. Commun. 2021, 12, 47683. 10.1038/s41467-021-20982-2 PMC7846803 33514748

[24] K. Takeda , T. Tanaka , W. Shi , M. Matsumoto , M. Minami , S.‐I. Kashiwamura , K. Nakanishi , N. Yoshida , T. Kishimoto , S. Akira , Nature 1996, 380, 627. 8602263 10.1038/380627a0

[25] Q. Tian , Y. Zhang , G. Wang , Z. Shen , Biomed. Pharmacother. 2017, 92, 1.28525794

[26] F. Neuenfeldt , J. C. Schumacher , R. Grieshaber‐Bouyer , J. Habicht , J. Schröder‐Braunstein , A. Gauss , U. Merle , B. Niesler , N. Heineken , A. Dalpke , M. M. Gaida , T. Giese , S. Meuer , Y. Samstag , G. Wabnitz , Cell Rep. 2022, 39, 110710. 35443164 10.1016/j.celrep.2022.110710

[27] L. J. O'Neil , A. Barrera‐Vargas , D. Sandoval‐Heglund , J. Merayo‐Chalico , E. Aguirre‐Aguilar , A. M. Aponte , Y. Ruiz‐Perdomo , M. Gucek , H. El‐Gabalawy , D. A. Fox , J. D. Katz , C. Carmona‐Rivera , Sci. Adv. 2020, 6, abd2688. 10.1126/sciadv.abd2688 PMC7608797 33115748

[28] M. F. Denny , S. Yalavarthi , W. Zhao , S. G. Thacker , M. Anderson , A. R. Sandy , W. J. McCune , M. J. Kaplan , J. Immunol. 2010, 184, 3284. 20164424 10.4049/jimmunol.0902199 PMC2929645

[29] J. M. Rorabaugh , T. Chalermpalanupap , C. A. Botz‐Zapp , V. M. Fu , N. A. Lembeck , R. M. Cohen , D. Weinshenker , Brain 2017, 140, 3023. 29053824 10.1093/brain/awx232 PMC5841201

[30] W. M. Freeze , S. J. Van Veluw , W. J. Jansen , D. A. Bennett , H. I. L. Jacobs , Alzheimers Dement. 2023, 19, 5023. 37095709 10.1002/alz.13096 PMC10593911

[31] I. Suarez‐Pereira , M. Llorca‐Torralba , Bravo , C. Camarena‐Delgado , C. Soriano‐Mas , E. Berrocoso , Biol. Psychiatry 2022, 91, 786. 35164940 10.1016/j.biopsych.2021.11.023

[32] J. Kruk , B. H. Aboul‐Enein , J. Bernstein , M. Gronostaj , Oxid. Med. Cell. Longev. 2019, 2019, 1270397. 31814865 10.1155/2019/1270397 PMC6877941

[33] X.‐Y. He , Y. Gao , D. Ng , E. Michalopoulou , S. George , J. M. Adrover , L. Sun , J. Albrengues , J. Daßler‐Plenker , X. Han , L. Wan , X. S. Wu , L. S. Shui , Y.u‐H. Huang , B. Liu , C. Su , D. L. Spector , C. R. Vakoc , L. Van Aelst , M. Egeblad , Cancer Cell 2024, 42, 474. 38402610 10.1016/j.ccell.2024.01.013 PMC11300849

[34] B. E. Cohen , D. Edmondson , I. M. Kronish , Am. J. Hypertens. 2015, 28, 1295. 25911639 10.1093/ajh/hpv047 PMC4612342

[35] F. C. C. M. Dobson , Annu. Rev. Biochem. 2017, 86, 35. 10.1146/annurev-biochem-061516-045115 28498720

[36] V. D'Aguanno , M. Ralli , M. Artico , F. Y. Russo , A. Scarpa , M. Fiore , P. Tirassa , C. Severini , M. de Vincentiis , A. Greco , Clin. Rev. Allergy Immunol. 2020, 59, 304. 31376044 10.1007/s12016-019-08759-4

[37] L. Dion‐Albert , A. Cadoret , E. Doney , F. N. Kaufmann , K. A. Dudek , B. Daigle , L. F. Parise , F. Cathomas , N. Samba , N. Hudson , M. Lebel , F. Aardema , L. Ait Bentaleb , J. Beauchamp , H. Bendahmane , E. Benoit , L. Bergeron , A. Bertone , N. Bertrand , F.‐A. Berube , P. Blanchet , J. Boissonneault , C. J. Bolduc , J.‐P. Bonin , F. Borgeat , R. Boyer , C. Breault , J.‐J. Breton , C. Briand , J. Brodeur , et al., Nat. Commun. 2022, 13, 164. 35013188 10.1038/s41467-021-27604-x PMC8748803

[38] F. Marino , A. Scanzano , L. Pulze , M. Pinoli , E. Rasini , A. Luini , R. Bombelli , M. Legnaro , M. de Eguileor , M. Cosentino , J. Leukoc. Biol. 2018, 104, 603. 29668114 10.1002/JLB.3A1017-398RR

[39] A. Clemente‐Moragón , E. Oliver , D. Calle , L. Cussó , M. Gómez , J. M. Pradillo , R. Castejón , N. Rallón , J. M. Benito , J. C. Fernández‐Ferro , J. Carneado‐Ruíz , M. A. Moro , J. Sánchez‐González , V. Fuster , M. Cortés‐Canteli , M. Desco , B. Ibáñez , Br. J. Pharmacol. 2023, 180, 459. 36181002 10.1111/bph.15963 PMC10100149

[40] D. Sharma , J. D. Farrar , Semin. Immunopathol. 2020, 42, 709. 33219396 10.1007/s00281-020-00829-6 PMC7678770

[41] T. K. Nayak , A. Bajpai , V. Patwa , R. L. Carter , N. Enjamuri , E. Gao , Y. K. Xiang , D. G. Tiley , bioRxiv 2023. 10.7150/thno.123651 PMC12905670 41695468

[42] M. A. Tanner , C. A. Maitz , L. A. Grisanti , Am. J. Physiol. Heart Circ. Physiol. 2021, 321, H633. 34415184 10.1152/ajpheart.00243.2021 PMC8816326

[43] A. Louveau , J. Herz , M. N. Alme , A. F. Salvador , M. Q. Dong , K. E. Viar , S. G. Herod , J. Knopp , J. C. Setliff , A. L. Lupi , S. Da Mesquita , E. L. Frost , A. Gaultier , T. H. Harris , R. Cao , S. Hu , J. R. Lukens , I. Smirnov , C. C. Overall , G. Oliver , J. Kipnis , Nat. Neurosci. 2018, 21, 1380. 30224810 10.1038/s41593-018-0227-9 PMC6214619

[44] L. Vong , P. M. Sherman , M. Glogauer , Methods Mol. Biol. 2013, 1031, 41. 23824885 10.1007/978-1-62703-481-4_5

[45] L. Yang , Q. Liu , X. Zhang , X. Liu , B. Zhou , J. Chen , D.i Huang , J. Li , H. Li , F. Chen , J. Liu , Y. Xing , X. Chen , S. Su , E. Song , Nature 2020, 583, 133. 32528174 10.1038/s41586-020-2394-6

[46] H. J. Selvadurai , J. O. Mason , PLoS One 2012, 7, e42572. 22880037 10.1371/journal.pone.0042572 PMC3411831

[47] S. M. Greenberg , A. Charidimou , Stroke 2018, 49, 491. 29335334 10.1161/STROKEAHA.117.016990 PMC5892842

