# Supplementary material for: Chronic Stress Exacerbates Cerebral Amyloid Angiopathy Through Promoting Neutrophil Extracellular Traps Formation
Source: Adv Sci (Weinh). 2024 Sep 26;11(43):2404096. doi: 10.1002/advs.202404096 (PMC11578353; doi:10.1002/advs.202404096)
Supplement: Supplementary file 1 — Supporting Information [file ADVS-11-2404096-s001.docx]

**Supplementary Material**

**Extended methods**

**Behavioral tests.** On day 29 of the CRS or CUMS protocol, both control and stress-exposed mice were subjected to open field test (OFT) and sucrose preference test (SPT). The OFT was performed in a nontransparent box (50 x 50 x 40 cm). Mice were placed in one of the corners of the arena at the start of each session. The center zone was set 17 x 17 cm in the middle of the arena. Mice were allowed to explore the arena freely for 5 minutes, then monitored using a USB 1080p web camera connected to a computerized video tracking system (VisuTrack). In SPT, mice were given access to two water bottles for 24 hours which were placed by bottles containing 1% sucrose in drinking water for another 24 hours for habituation. Before formal test, mice were subjected to food and water deprivation overnight, and were able to access a water bottle and a bottle containing 1% sucrose solution for 1 hour. Sucrose preference was assessed as follows (based on weight of bottles): (sucrose consumption (g)/ total fluid consumption (g)) x 100%. After treatment of PADI4 inhibitor, CXCR2 inhibitor or vehicle, CAA mice were subjected to Novel object recognition (NOR) and Morris water maze (MWM). The NOR was performed in a nontransparent box (50 x 50 x 40 cm). The mouse was allowed to explore and habituated to two identical objects for 10 minutes. Then one of the training objects was replaced with a novel object with a different shape and color but roughly the same height and volume, and mice was allowed to explore the two objects for 10 minutes. All the mice finished the test inside 2 hours after the habituation progress. Recognition (RI) and discrimination indices (DI) were used to evaluate their performance. RI = new object exploration time / total exploration time. DI = (new object exploration time - familiar object exploration time) / total exploration time. In MWM, mice were placed in a pool (diameter: 120 cm; depth: 40-50 cm) of water (22°C ± 2°C) and trained to find a hidden platform (diameter: 15 cm) submerged 1.5 cm below the water surface for four consecutive days. In the spatial learning phase, mice were placed into the water at designated positions of 4 quadrants. If the mice failed to get to the platform within 60s, it was guided to the platform, and was allowed to remain on the platform for 10s. The escape latency (time to reach the platform) and the travel distance before they reached the platform were used to evaluate acquisition of the learning task. Each trial was repeated four times with 1-min intervals, which comprised 1 set. The releasing location was changed in each trial. The mice were trained for at least one set a day up to 4 days (training session). Daily training was started at a fixed time, and a second set was performed 1 h after the first set. After the training session, the mice were allowed to swim for 60s in the absence of the platform from the point farthest from the platform’s former location. Memory for the platform location was assessed by quantifying the latency before they get to the platform firstly and the time of crossing the place and in which the platform had been previously placed in the training session (probe test).

**Bulk-RNA sequencing.** A total of 5×10^6^ BMDN were collected at the end of treatment. BMDNs were isolated and stored in TRIzol Reagent at the temperature of -80℃. BMDN samples were then sent to Novogene Technology Co., Ltd. (Beijing, China) for high-throughput sequencing analysis.

**Scanning Electron Microscopy (SEM).** Cortex was cut and harvested using sharp blade quickly and washed with PBS gently, then immediately fixed by electron microscopy fixative (Servicebio, G1102) for 2h at room temperature, then postfixed in 1% OsO4 in 0.1 M PB (pH 7.4) for 2 h, dehydrated with a graded ethanol series (25-100%), and critical point dried from CO_2_. The dried tissue was attached on metallic stubs with carbon stickers and sputter-coated with gold for 30s. Sample was observed and imaged in a HITACHI scanning electron microscope at 5 kV.

**Immunofluorescence staining.** The following primary antibodies were used: rabbit anti-MBP (Proteintech 10458-1-AP, 1:500), mouse anti-Aβ40 monoclonal antibodies (R&D MAB96181R-SP, 1:500), rat anti-CD31 (Bioscience 550274, 1:50), mouse anti-Ly6G (Proteintech 65140-1-IG, 1:500), rabbit anti-ZO1 (Invitrogen 617300, 1:500), chicken anti-NFH (Biolegend 822601, 1:500), rabbit anti-CitH3 (abcam ab281584 1:500), rabbit anti-Neutrophil Elastase (abcam ab131260, 1:500), rabbit anti-S100A9 (Proteintech 26992-1-AP, 1:500), mouse anti-8-Hydroxyguanosine (GeneTex GTX41980, 1:500), rabbit anti-pSTAT6 (Cell Signaling Technology 9361T, 1:500). The following secondary antibodies were applied: anti-rat secondary antibody conjugated with Cy3 (Jackson ImmunoResearch Laboratories 112-545-003, 1:1000), anti-rabbit secondary antibody conjugated with Cy3 (Jackson ImmunoResearch Laboratories 111-165-003, 1:1000), anti-rabbit secondary antibody conjugated with 488 (Jackson ImmunoResearch Laboratories 111-545-003,1:1000), anti-mouse secondary antibody conjugated with Alexa Fluor 488 (Invitrogen A-11059, 1:1000), and anti-chicken secondary antibody conjugated with FITC (Jackson ImmunoResearch Laboratories 303-095-003,1:1000).

**Flow cytometric analysis.** The following antibodies were used: APC-conjugated anti mouse CD45 (Biolegend 103112, clone: 30-F11, 1:400), BV421-conjugated anti mouse F4/80 (Biolegend 123132, clone: 8M8, 1:400), APC/CY7-conjugated anti mouse Ly6G (Biolegend 108424, clone: RB8-8C5, 1:400), FITC-conjugated anti mouse CD3 (Biolegend 100204, clone: 17A2, 1:400), PE-conjugated anti mouse CD19 (Biolegend 152408, clone: 1D3/CD19, 1:400). Percp/CY5.5-conjugated anti mouse pSTAT6 (Biolegend 686010, clone: A15137E, 1:200).

**Western blot.** The following primary antibodies were used: rabbit anti-pSTAT6 (Cell Signaling Technology 9361T, 1:1000), rabbit anti-STAT6 (Cell Signaling Technology 5397T, 1:1000), rabbit anti-pSTAT3 (Cell Signaling Technology 9145T, 1:1000), rabbit anti-STAT3 (Proteintech 10253-2-AP, 1:1000), rabbit anti-pSTAT1 (Cell Signaling Technology 9167S, 1:1000), rabbit anti-STAT1 (Cell Signaling Technology 9172T, 1:1000), mouse anti-β-actin (Proteintech 66009-1-Ig, 1:1000).

**Cell viability analysis.** BMDN viability was assessed with Lactate Dehydrogenase (LDH) assay (Invitrogen, C20300) and LIVE/DEAD Viability analysis (ThermoFisher, L7013) based on SYTO10 (green, outlined both lived and dead cells) and Ethidium homodimer-2 (red, indicating dead or injured cells) staining according to instructions from manufacturers.

**Single-cell RNA-sequencing analysis.** Downstream analysis was performed with the software of R (version 4.4.0). After filtering and normalization, samples were integrated directly, and performed dimensionality reduction and clustering using the Seurat V4 in R. Variable genes (genes = 4000) were selected through FindVariableFeatures() function and those variable genes were assigned onto a low-dimensional subspace using principal component analysis through RunPCA() function. Seurat functions FindNeighbors() and FindClusters() were used to assign subtypes with the top 50 principal components. To visualize the results, tSNE analysis was performed with Harmony embeddings. Differentially expressed genes were calculated with the FindMarker() function in Seurat (Wilcoxon rank-sum test). We queried the top differentially expressed genes of each cluster in a literature search to annotate the clusters. For gene ontology (GO) analysis, differentially expressed genes for each cluster were separated into up- and downregulated and separately the *enrichGO* function from the clusterProfiler package was used with a gene set size set between 10 and 500 genes and *P* values adjusted using the Benjamini-Hochberg correction.

**Supplementary figures**

**
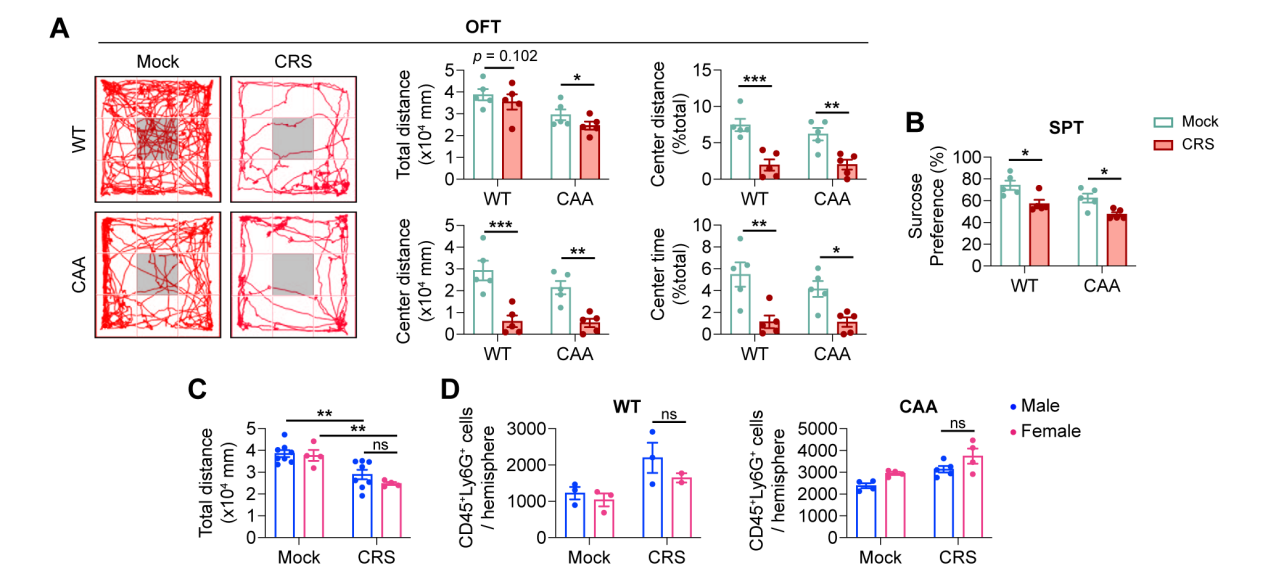
**

**Figure S1. Chronic restraint stress induces anxiety-like behavior in WT and CAA mice. (A-B)** WT or CAA mice (age = 12w) were stimulated with chronic restraint stress (CRS) for 4 weeks, and subjected to OFT and SPT to ensure psychological alteration after chronic stress. *N* = 5 mice in each group. **P* < 0.05, ***P* < 0.01, ****P* < 0.001; by one-way *ANOVA*. (**A)** Representative traces of mice’s paths in the OFT, and quantification of the total travel distance, the traveled distance or time in the center zone as a percentage of the total traveled distance or time after CRS. (**B**) SPT. (**C**) Data of OFT in (**A-B**) was shown in males and females separately to elucidate influence of sex on psychological alteration. *N* = 8 in Mock-Male and CRS-Male groups, and *N* = 4 in Mock-Female and CRS-Female groups. ***P* < 0.01; by one-way *ANOVA*. (**D**) Data of **Figure 1E** was shown in males and females separately to elucidate influence of sex on neutrophil infiltration. *N* = 2 in WT-CRS-Female group, *N* = 3 in WT-Mock-Male, WT-Mock-Female and WT-CRS-Male groups, *N* = 4 in CAA-Mock-Male, CAA-Mock-Female and CAA-CRS-Female groups, and *N* = 5 in CAA-CRS-Male group, by one-way *ANOVA*.

**
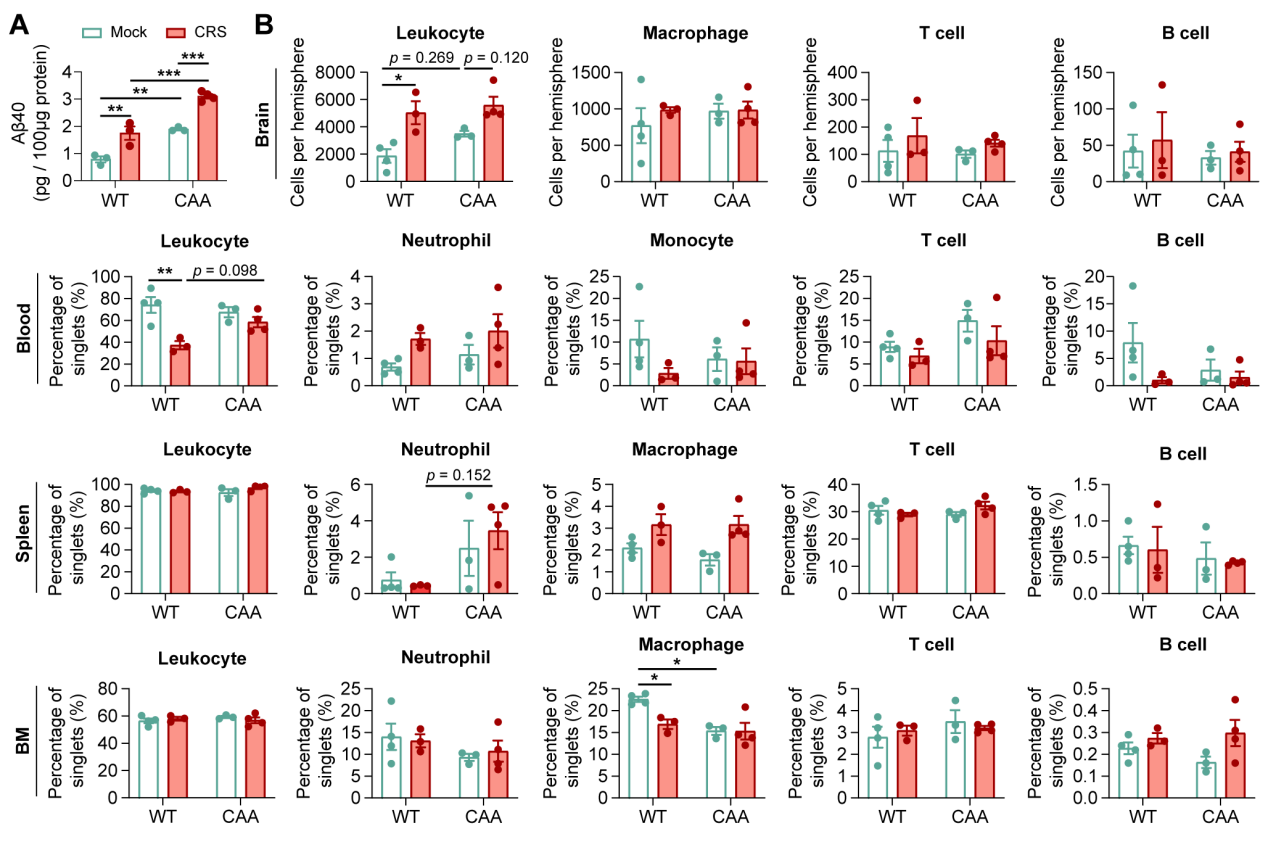
**

**Figure S2. Leukocyte constitution in the context of chronic stress.** WT or CAA mice (age = 12w) were stimulated with chronic restraint stress (CRS) for 4 weeks. (**A**) Concentration of Aβ40 in mice brain was assessed with ELISA. *N* = 3-4 mice in each group. ***P* < 0.01, ****P* < 0.001; by one-way *ANOVA*. (**B**) Leukocyte constitution in brain, blood, spleen and bone marrow (BM) was assessed with flow cytometry. Cell count of total leukocyte (CD45^+^), neutrophil (CD45^+^F4/80^-^Ly6G^+^, data shown in **Figure 1E**), macrophage (CD45^+^F4/80^+^), T cell (CD45^+^CD3^+^) and B cell (CD45^+^CD19^+^) per brain hemisphere or the respective percentage among singlets in blood, spleen and bone marrow were calculated. *N* = 3-4 mice in each group. **P* < 0.05, ***P* < 0.01; by one-way *ANOVA*.


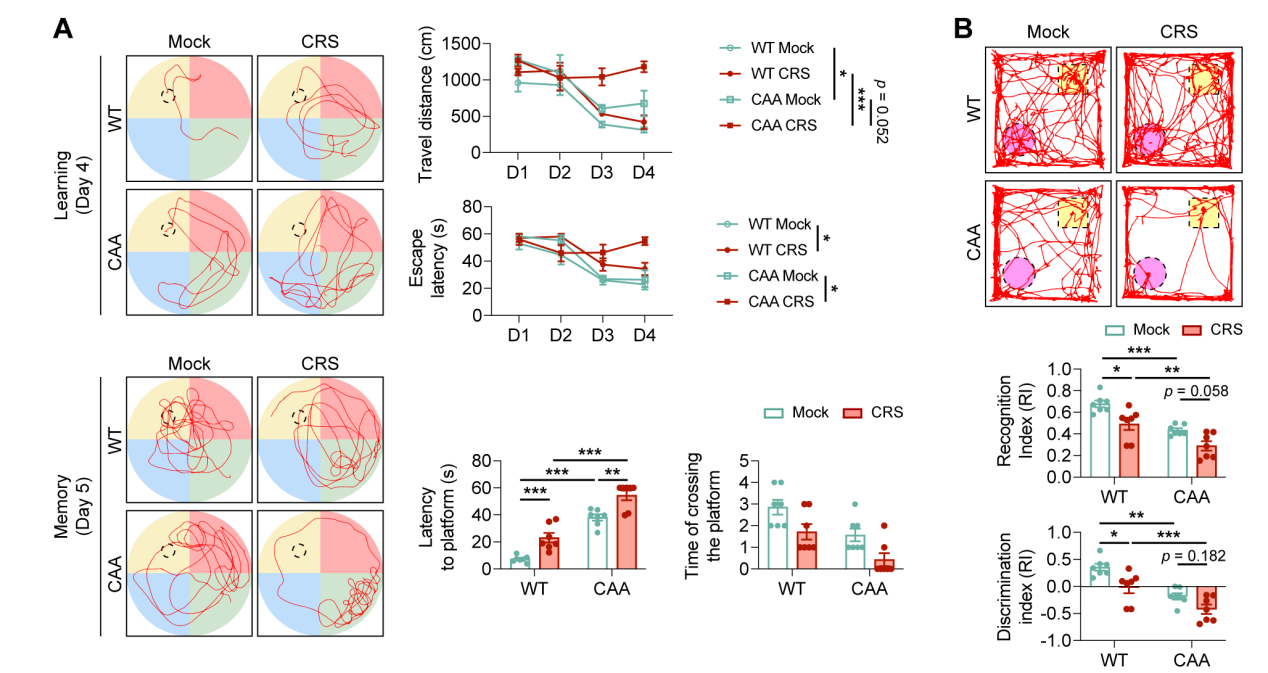


**Figure S3. Chronic restraint stress exacerbates cognitive impairment in CAA mice.** (**A**-**B**) WT or CAA mice (age = 12w) were stimulated with chronic restraint stress (CRS) for 4 weeks, and subjected to Morris water maze (**A**) and Novel object recognition (**B**) to evaluate the cognitive alteration. *N* = 7 mice in each group. **P* < 0.05, ***P* < 0.01, ****P* < 0.001; by two-way *ANOVA* in (**A, upper**), and by one-way *ANOVA* in (**A, lower, B)**.


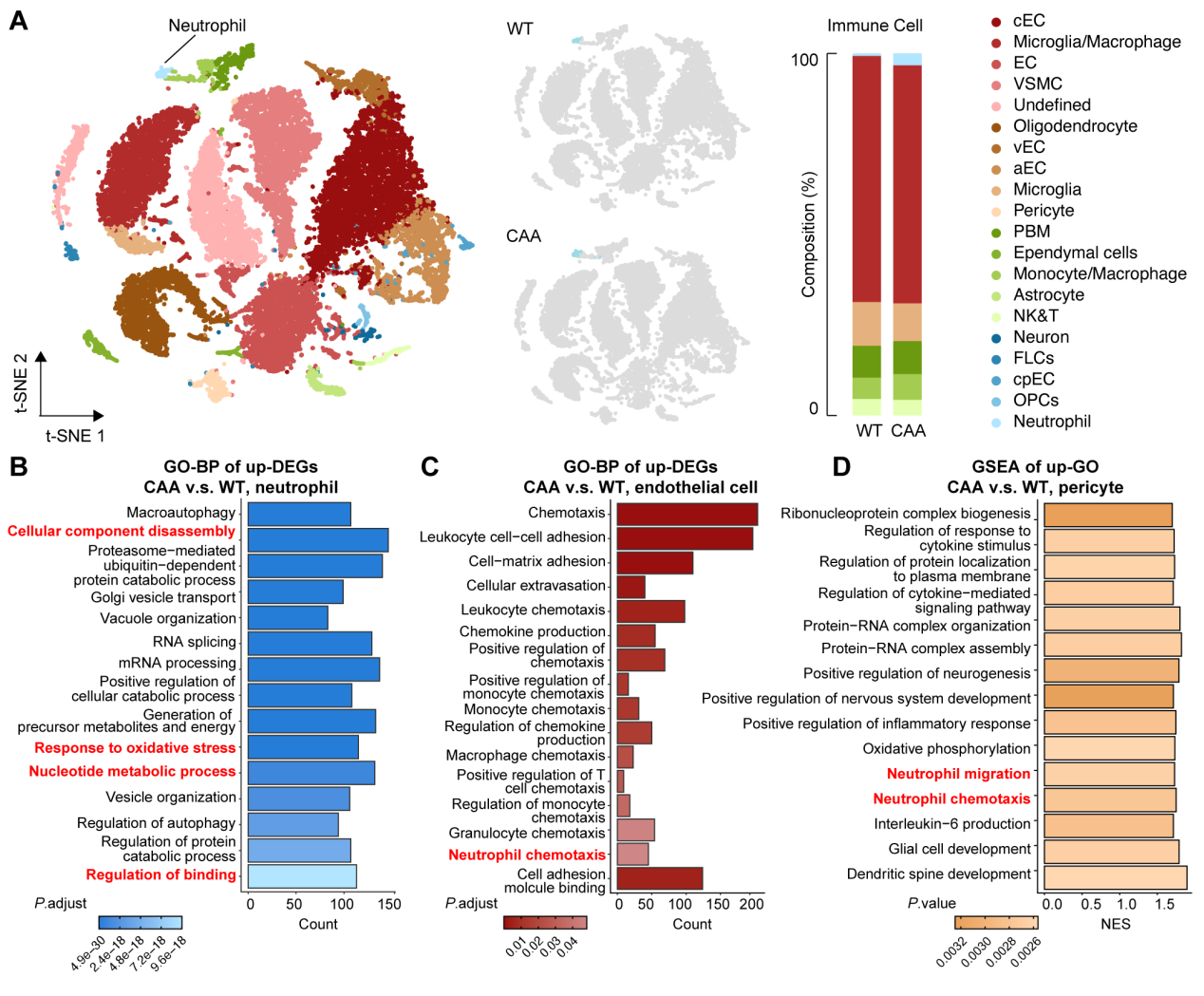


**Figure S4. Single-cell RNA sequencing of CAA or WT brains.** (**A**) (**Left**) t-SNE of 20 major cell populations identified in the brain of CAA and sex-matched WT mice (24w of age). Neutrophil clusters in the WT group (**Top middle**) and the CAA group (**Bottom middle**) were marked. (**Right**) Stacked bar graphs of immune cell type proportions in the WT and CAA groups. (**B**) Top 15 significant GO-BP terms in neutrophil. (**C**) GO-BP analysis of the up-regulated DEGs in endothelial cell. (**D**) Top 15 significant GSEA-GOBP terms in pericyte.

**
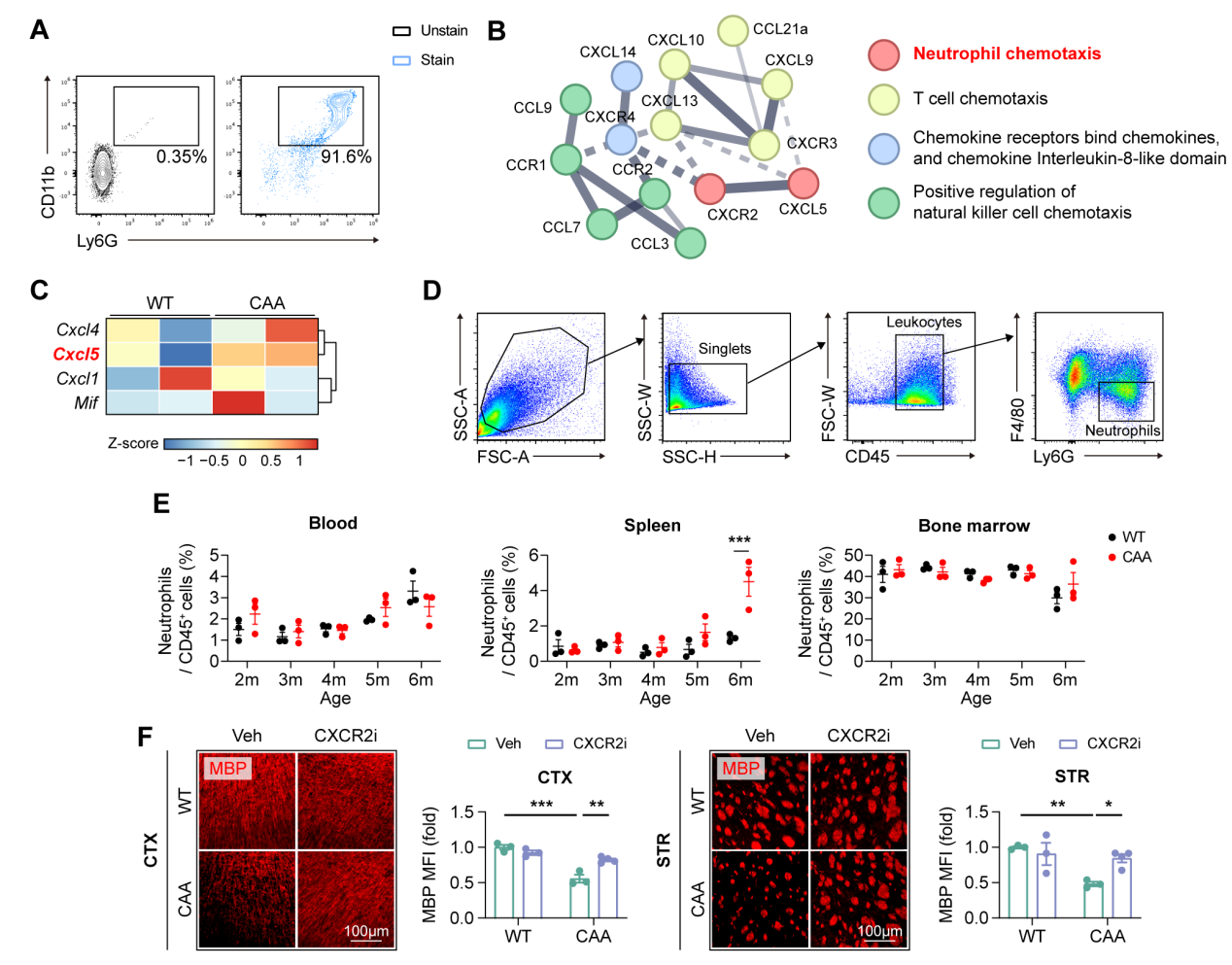
**

**Figure S5. Brain-targeting chemotaxis of neutrophils in CAA.** (**A**) Purity of BMDN was verified by flow cytometry. (**B-C**) Brain tissue of WT and CAA mice was subjected to bulk RNAseq. *N* = 2 mice in each group. (**B**) PPI network of up-regulated DEGs of chemotaxis associated genes in CAA brain compared to WT. The edges indicate that the proteins are part of a physical complex, and line thickness indicates the strength of data support. (**C**) Gene expression of CXCR2 responsive chemokines in WT and CAA brains. (**D-E**) Percentage of neutrophils (CD45^+^F4/80^-^Ly6G^+^) among leukocytes (CD45^+^) in blood, spleen and bone marrow of mice was calculated with flow cytometry. (**D**) Gating strategy. (**E**) Percentage of neutrophils among leukocytes in the periphery of WT and CAA mice at indicated age. *N* = 3 mice in each group. ****P* < 0.001; by two-way *ANOVA*. (**F**) CAA or WT mice (age = 12w) were treated with CXCR2i (SB225002, i.p., 2mg/kg, 3 weeks, 6 days each), and equal volume of PBS (vehicle, Veh) was injected to the control groups. All were sacrificed at 4w after treatment onset. Equal volume of PBS (vehicle, Veh) was injected to the control groups. White matter integrity in cortex (CTX) and striatum (STR) was assessed with MBP staining. *N* = 3-4 in each group. **P* < 0.05, ***P* < 0.01, ****P* < 0.001; by one-way *ANOVA*.


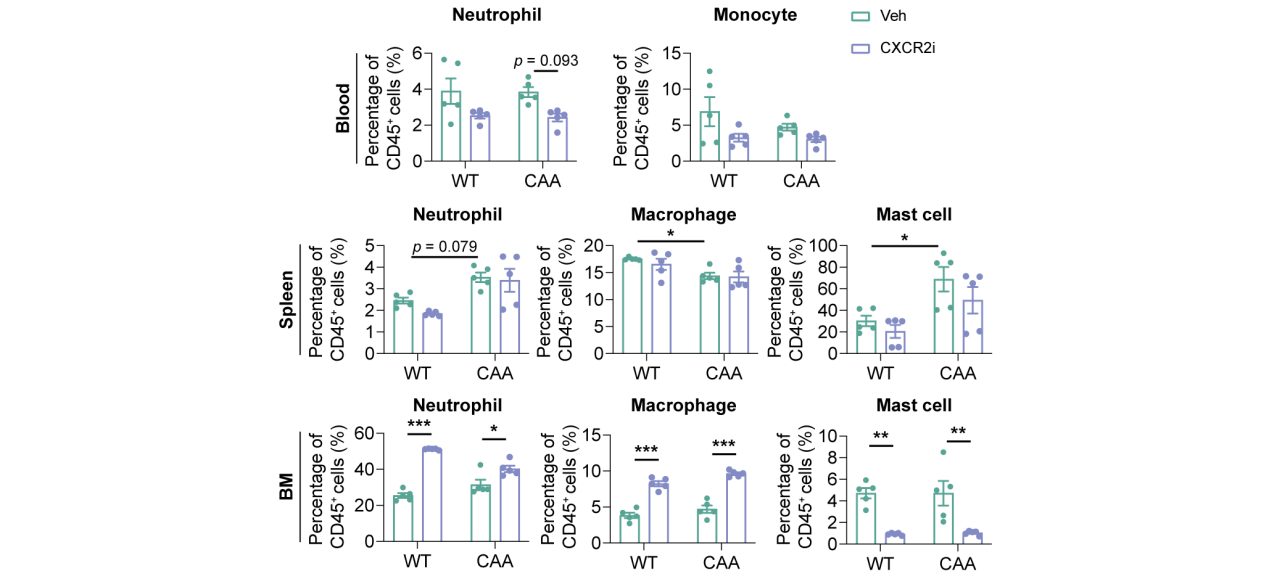


**Figure S6. The effect of SB225002 on periphery immune cells.** CAA or WT mice (age = 12w) were treated with CXCR2i (SB225002, i.p., 2mg/kg, 3 weeks, 6 days each) and sacrificed at 4w after treatment onset. Equal volume of PBS (vehicle, Veh) was injected to the control groups. Percentage among CD45^+^cell of neutrophil (CD45^+^F4/80^-^Ly6G^+^, macrophage (CD45^+^F4/80^+^Ly6G^-^) and mast cell (CD45^+^CD117^+^) in blood, spleen and bone marrow were calculated. *N* = 5 in each group. **P* < 0.05; by one-way *ANOVA*.


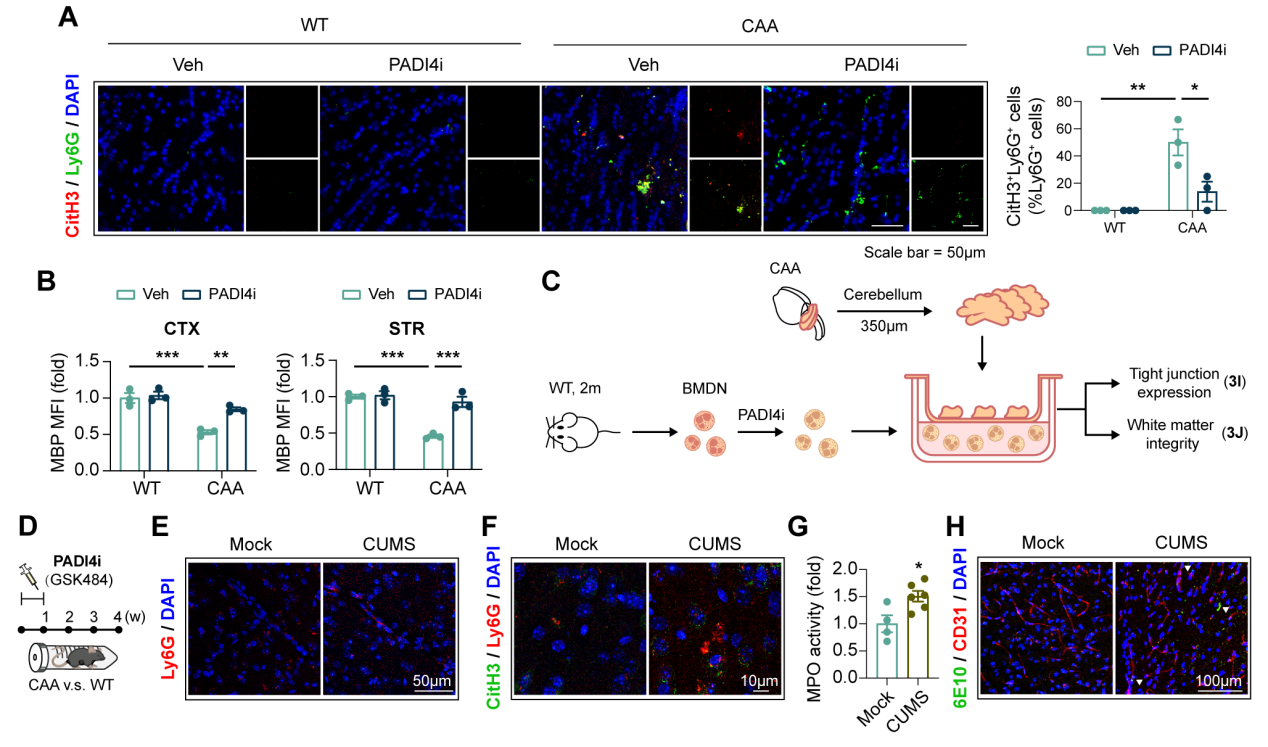
**Figure S7. PADI4 inhibition ameliorates white matter injury in CAA through inhibiting NET formation.** CAA or WT mice (age = 12w) were treated with PADI4i (GSK484, i.p., 4mg/kg, for 1w) and sacrificed at 4w after treatment onset. Equal volume of PBS (Veh) was injected to the control groups. *N* = 3 mice in each group. (**A**) NET-inhibiting efficacy of PADI4i treatment was validated with immunostaining of Ly6G and CitH3. *N* = 3 mice in each group. **P* < 0.05, ***P* < 0.01; by one-way *ANOVA*. (**B**) Quantification of MBP MFI in cortex (CTX) and striatum (STR) in **Figure 3H** was calculated. *N* = 3 mice in each group. ***P* < 0.01, ****P* < 0.001; by one-way *ANOVA*. (**C**) Experimental design of **Figure 3H-J**. (**D**) Experimental design of **Figure 4D-F**. (**E**-**H**) WT mice (age = 8w) were subjected to CUMS for 4w. *N* = 4 in Mock group, and *N* = 6 in CUMS group. (**E**) Neutrophil infiltration in brains of CUMS mice was confirmed with immunostaining of Ly6G. (**F**) NETs accumulation in brains of CUMS mice was confirmed with immunostaining of Ly6G and CitH3. (**G**) MPO activity of mice brain was detected by ELISA. **P* < 0.05; by two-tailed Student’s *t* test. (**H**) Aβ deposition in brains of CUMS mice was confirmed with immunostaining of 6E10 and CD31. White arrows head emphasize Aβ.


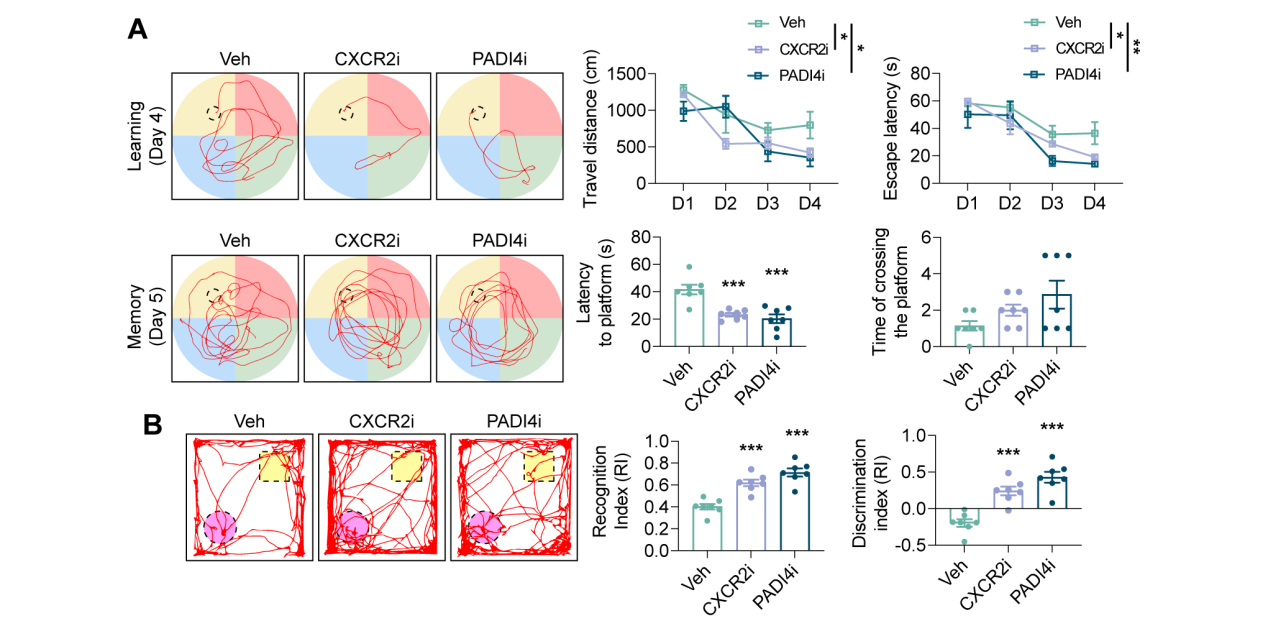


**Figure S8. Inhibition of neutrophil chemotaxis or NETosis ameliorates cognitive decline in CAA mice.** (**A**-**B**) CAA mice (age = 12w) were treated with CXCR2i (SB225002, i.p., 2mg/kg, 3 weeks, 6 days each) or PADI4i (GSK484, i.p., 4mg/kg, for 1w) and subjected to Morris water maze (**A**) and Novel object recognition (**B**) to evaluate the cognitive alteration at 4w. *N* = 7 mice in each group. **P* < 0.05, ***P* < 0.01, ****P* < 0.001; by two-way *ANOVA* in (**A, upper**), and by one-way *ANOVA* in (**A, lower, B)**.


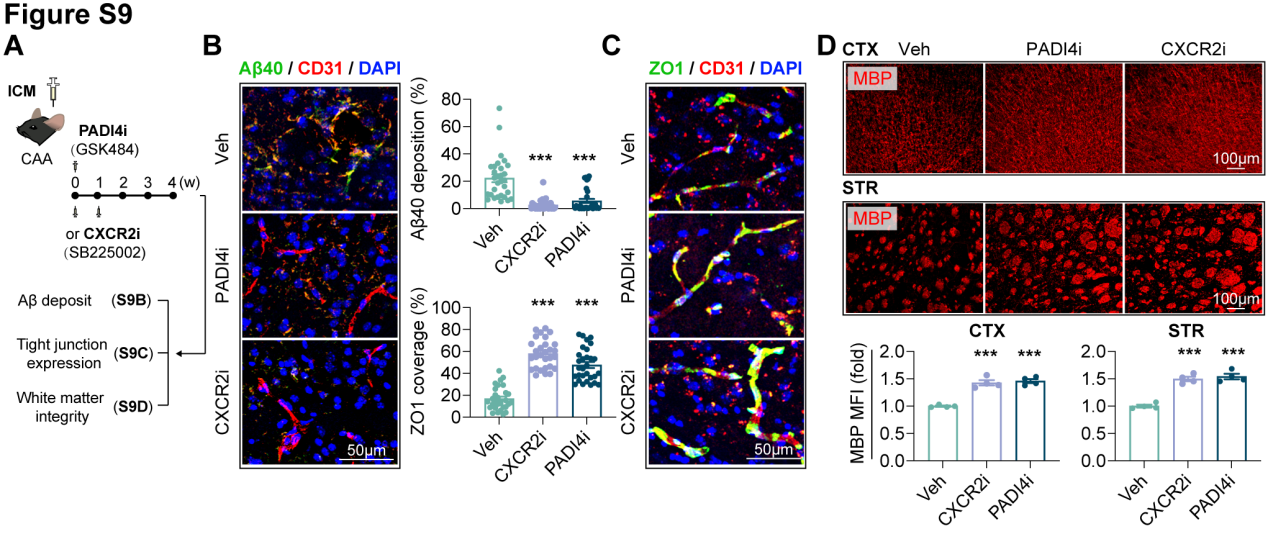


**Figure S9. Suppression of neutrophil chemotaxis or NET formation in central nervous system improves CAA conditions.** (**A**-**C**) CAA mice (age = 16w) were treated with CXCR2i (SB225002, 6μg in 3μl, once a week for 2w) or PADI4i (GSK484, i.c.m., 8μg in 2μl) and sacrificed at 4w after treatment onset. PBS (vehicle, Veh) was injected to the control groups. *N* = 4 mice in each group. (**A**) Experimental design. (**B**) Coverage of Aβ40 fluorescence in the range of blood vessels (outlined with CD31) in cortex was calculated. A total of 30 blood vessels in each group were analyzed respectively. ****P* < 0.001; by one-way *ANOVA*. (**B**) Coronal brain sections were subjected to immunostaining of ZO1 and CD31. ZO1 coverage in 30 blood vessels in each group was analyzed respectively. ****P* < 0.001; by one-way *ANOVA*. (**H**) White matter integrity in cortex (CTX) and striatum (STR) was assessed with MBP staining. ****P* < 0.001; by one-way *ANOVA*.


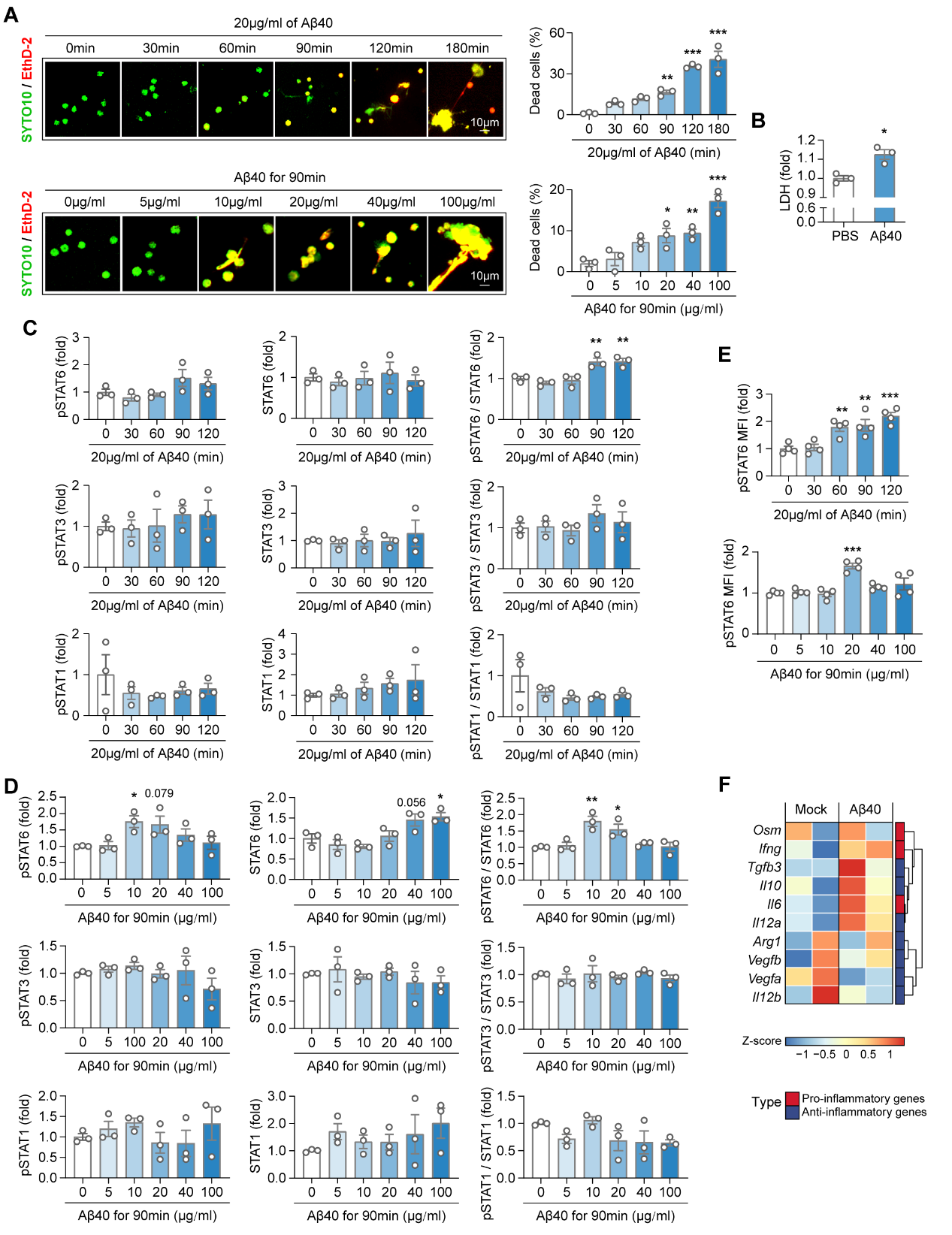


**Figure S10. Aβ40 elicits NET formation through activating STAT6 signaling.** (**A**) BMDN were treated with 20μg/ml of Aβ40 for various time period (**upper**) or Aβ40 at various concentration for 90min (**lower**). SYTO10 and Ethidium homodimer-2 (EthD-2) staining was performed to evaluate cell death. The percentage of dead cells (SYTO10^+^EthD2^+^) was calculated. Experiments were repeated for 3 times. **P* < 0.05, ***P* < 0.01, ****P* < 0.001; compared with 0min or 0μg/ml group; by one-way *ANOVA*. (**B**) BMDN were treated with Aβ40 (20μg/ml, 90min). LDH released in the culture medium was assessed with ELISA. Experiments were repeated for 3 times. **P* < 0.05; by Student’s *t* test. (**C-D**) BMDN were treated with 20μg/ml of Aβ40 for various time period (**C**) or Aβ40 at various concentration for 90min (**D**) then subjected to western blot. Experiments were repeated for 3 times. **P* < 0.05, ***P* < 0.01; compared with 0min or 0μg/ml group; by one-way *ANOVA*. (**E**) BMDN were treated with 20μg/ml of Aβ40 for various time period (**upper**) or Aβ40 at various concentration for 90min (**lower**). pSTAT6 MFI as assessed with flow cytometry was calculated. Experiments were repeated for 4 times. ***P* < 0.01, ****P* < 0.001; compared with 0min or 0μg/ml group; by one-way *ANOVA*. (**F**) BMDN with or without Aβ40 treatment (20μg/ml, 90min) were subjected to bulk RNAseq. Expression of inflammatory genes was displayed. *N* = 2 in each group.


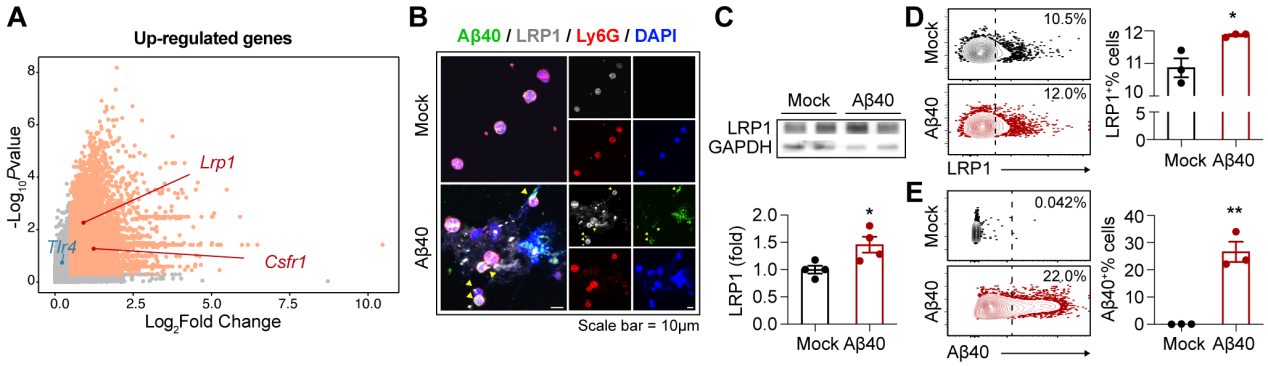


**Figure S11. Aβ is internalized via LRP1 to induce NETs formation.** (**A**) Up-regulated differential expressed genes (DEGs) of neutrophil in brains of CAA and WT mice (age = 16w). Genes of receptors associated with Aβ have been labeled, with those marked in red indicating statistical significance and the one in blue indicating no significance. (**B**) BMDN isolated from WT mice were treated with 20μg/ml of Aβ40-FAM for 1.5h then subjected to immunostaining of LRP1 and Ly6G. Yellow arrowhead emphasize colocalization of Aβ40-FAM and LRP1. (**C**) BMDN were treated with 20μg/ml of Aβ40 for 1.5h then subjected to western blot. Experiments were repeated for 4 times. **P* < 0.05; by Student’s *t* test. (**D**-**E**) BMDN were treated with 20μg/ml of Aβ40-FAM for 1.5h. Percentage of LRP1^+^ (**D**) or Aβ40^+^ (**E**) cells as assessed with flow cytometry was calculated. Experiments were repeated for 3 times. **P* < 0.05, ***P* < 0.01; by Student’s *t* test.


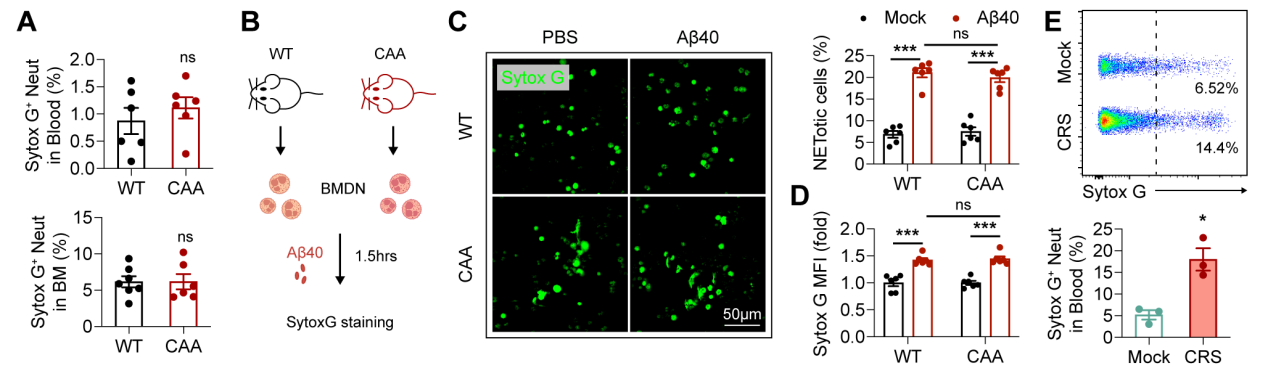


**Figure S12. Neutrophil from CAA mice exhibits a comparable capacity for NETs production.** (**A**) Neting neutrophil (Neut) in blood and bone marrow (BM) of CAA and WT mice was assessed by Sytox green staining with flow cytometry. *N* = 6 in each group. by Student’s *t* test. (**B**-**D**) BMDN isolated from CAA or WT mice were treated with 20μg/ml of Aβ40 for 1.5h then subjected to Sytox green staining. *N* = 6 in each group. (**B**) Experimental design. (**C**) BMDN were fixed and subjected to fluorescence microscopy. ****P* < 0.001; by one-way *ANOVA*. (**D**) Sytox G MFI was assessed with a plate reader and normalized to the non-treated group. ****P* < 0.001; by one-way *ANOVA*. (**E**) Neting neutrophil (Neut) in blood of WT mice received CRS was assessed by Sytox green staining with flow cytometry. *N* = 3 in each group. **P* < 0.05; by Student’s *t* test.


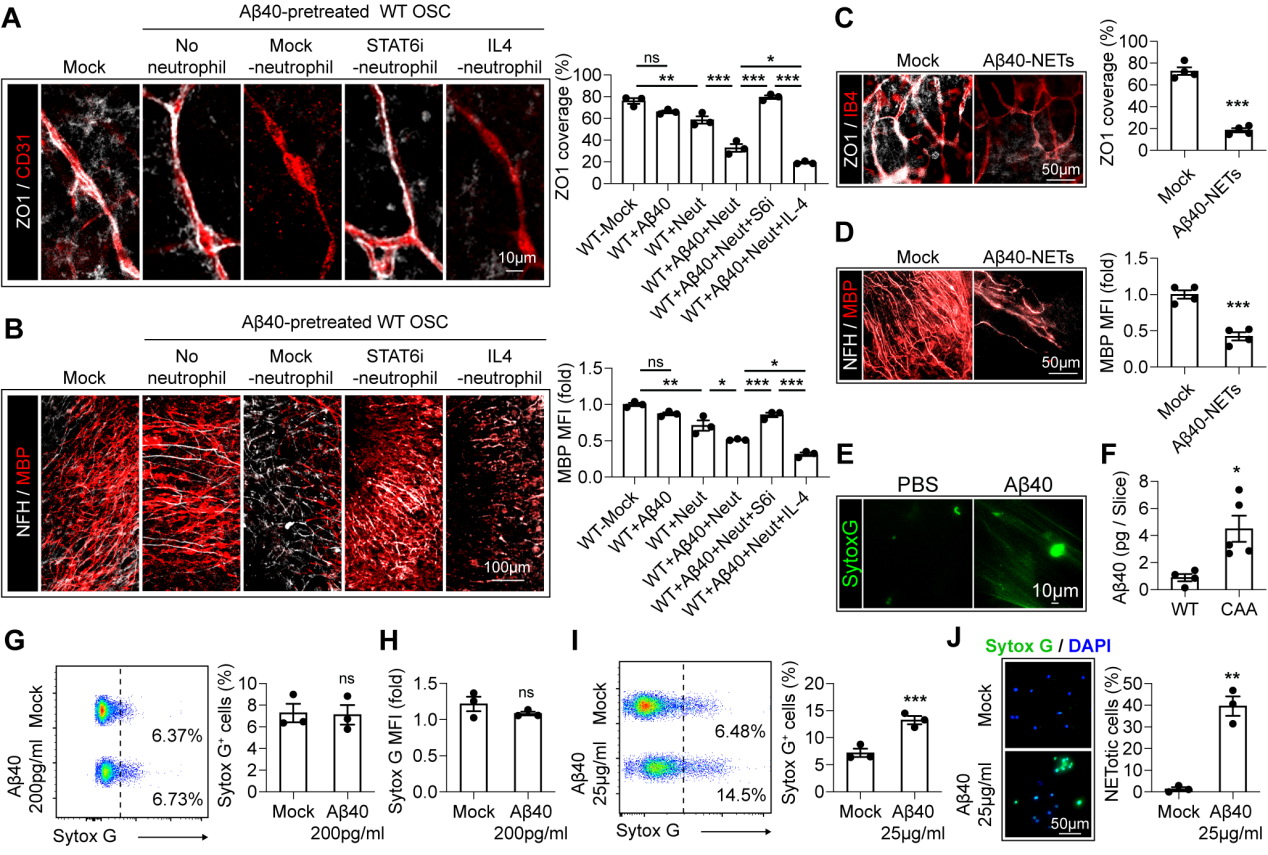


**Figure S13. STAT6 activation enhances the injurious effects of neutrophils to brain tissue.** (**A-B**) OSCs were prepared with the cerebellum of WT mice. WT OSCs were coated with Aβ40 (20μg/ml overnight) to simulate CAA pathology. BMDN were extracted from WT mice then pretreated with STAT6i (1μM, STAT6i-neutrophil) or IL-4 (20ng/ml, IL4-neutrophil) for 1h. BMDN were then cocultured with the OSCs through transwell for 4h (5×10^4^ BMDN per culture system with 3 brain slices). Experiments were repeated for 3 times. (**A**) ZO1 and CD31 were co-stained to evaluate the tight junction loss of OSC after co-cultured with BMDN. (**B**) White matter integrity was assessed with immunostaining of MBP and NFH. Experiments were repeated for 3 times. **P* < 0.05, ***P* < 0.01, ****P* < 0.001; by one-way *ANOVA*. (**C-D**) OSCs were prepared with the cerebellum of WT mice and treated with Aβ40-induced NETs for 48h. Tight junction loss was assessed with immunostaining of ZO1 and CD31(**C**) while white matter integrity was assessed with staining of MBP and NFH (**D**). Experiments were repeated for 4 times. ****P* < 0.001; by Student’s *t* test. (**E**) BMDN was collected and subjected to Sytox G staining to reveal the extracellular DNA traps after being cocultured with Aβ40-pretreated OSCs. (**F**) Concentration of Aβ40 in CAA- or WT-OSC were measured. *N* = 4-5 in each group.**P* < 0.05; by Student’s *t* test. (**G-H**) BMDN from WT mice were exposed to Aβ40 at a physiological concentration of 200pg/ml and assessed by Sytox G staining with flow cytometry (**G**) and a plate reader and normalized to the non-treated group (**H**). by Student’s *t* test. (**I-J**) BMDN isolated from WT mice were exposed to Aβ40 at a concentration of 25μg/ml corresponding to the level measured in the brains of CAA mice as shown in **Figure S2A** for 1.5h then assessed by Sytox green staining with flow cytometry (**I**) and fluorescence microscopy (**J**). Experiments were repeated for 3 times. ****P* < 0.001; by Student’s *t* test.


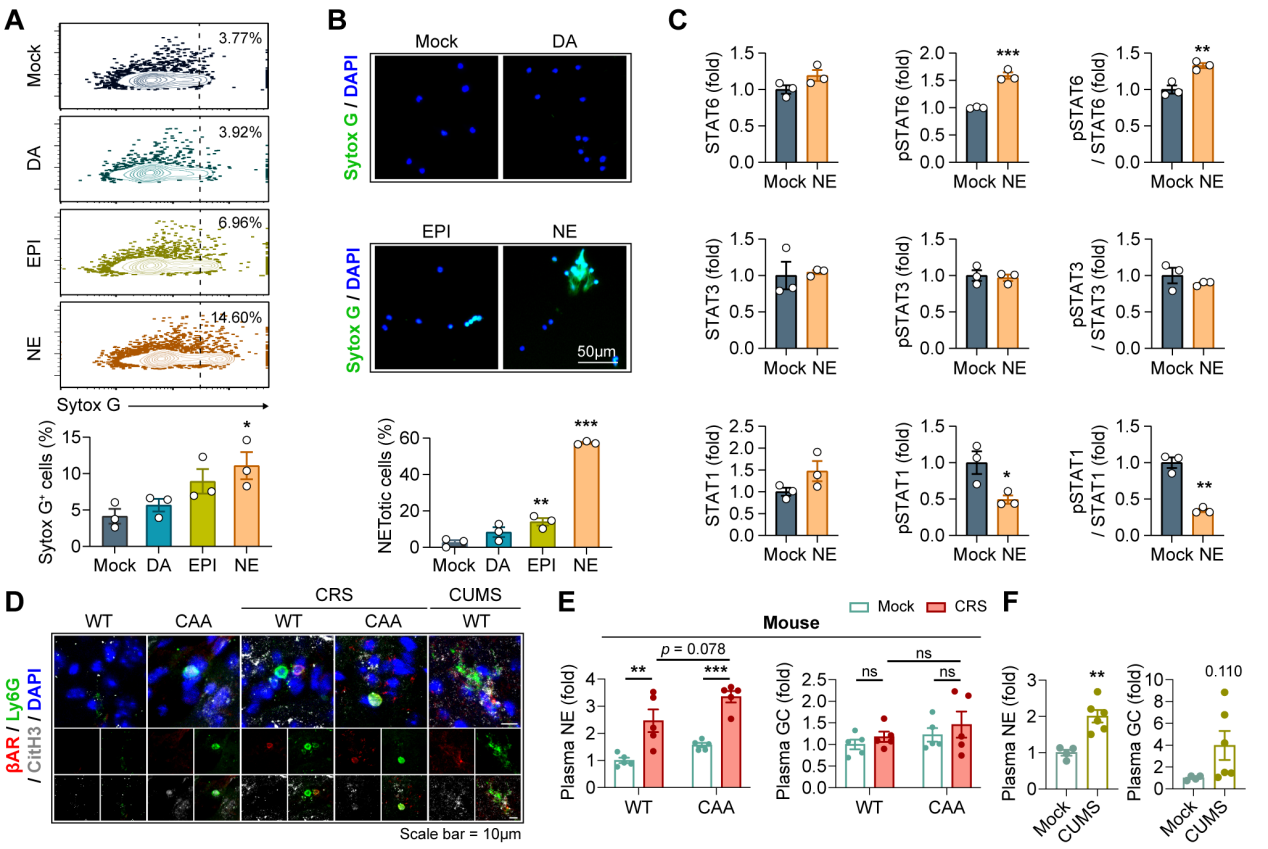


**Figure S14. Norepinephrine elicits NET formation through activating STAT6 signaling.** (**A-B**) BMDN were extracted from WT mice then treated with Norepinephrine (NE, 0.1μM), Epinephrine (EPI, 0.1μM) and Dopamine (DA, 0.1μM) for 2h. Sytox G was applied to reveal the extracellular DNA traps. Percentage of NETotic BMDN (Sytox G^+^) was assessed with flow cytometry (**A**) or fluorescence microscopy (**B**). Experiments were repeated for 3 times. **P* < 0.05, ***P* < 0.01, ****P* < 0.001; compared with Mock group; by one-way *ANOVA*. (**C**) BMDN treated with NE (0.1μM, 2h) were subjected to western blot to evaluate activation of STAT signaling. Experiments were repeated for 3 times. Representative gel plots are displayed in **Figure 7H**. **P* < 0.05, ***P* < 0.01, ****P* < 0.001; by Student’s *t* test. (**D**) Up-regulated expression of β-adrenergic receptor on brain-infiltrating neutrophils after chronic stress was validated with immunostaining of βΑR, Ly6G and CitH3. (**E**) Concentration of Norepinephrine (NE) and Glucocorticoid (GC) in plasma of mice received CRS was quantified by ELISA. *N* = 5 in each group. ***P* < 0.01, ****P* < 0.001; by one-way *ANOVA*. (**F**) Concentration of Norepinephrine (NE) and Glucocorticoid (GC) in plasma of mice received CUMS was quantified by ELISA. *N* = 4 in Mock group, and *N* = 6 in CUMS group. ***P* < 0.01; by Student’s *t* test.

**Table S1. Clinic characteristics of the individuals recruited in the study**

| **Clinical Characteristics** | **Healthy controls** | **CAA** |
| --- | --- | --- |
| *N* | 27 | 26 |
| Age, y | 65.96±8.40 | 73.85±8.72 |
| Male, n (%) | 9 (33.3%) | 15 (57.7%) |
| Female, n (%) | 18 (66.7%) | 11 (43.3%) |

Results were presented as mean ± SEM for normally distributed continuous variables and median (quartiles) for skewedly distributed continuous variables
